# Unveiling Palmitoyl Thymidine Derivatives as Antimicrobial/Antiviral Inhibitors: Synthesis, Molecular Docking, Dynamic Simulations, ADMET, and Assessment of Protein–Ligand Interactions

**DOI:** 10.3390/ph18060806

**Published:** 2025-05-27

**Authors:** Sarkar M. A. Kawsar, Samiah Hamad Al-mijalli, Gassoumi Bouzid, Emad M. Abdallah, Noimul H. Siddiquee, Mohammed A. Hosen, Mabrouk Horchani, Houcine Ghalla, Hichem B. Jannet, Yuki Fujii, Yasuhiro Ozeki

**Affiliations:** 1 Laboratory of Carbohydrate and Nucleoside Chemistry, Department of Chemistry, Faculty of Science, University of Chittagong, Chittagong 4331, Bangladesh; m.a.hossain1996@gmail.com; 2 Department of Biology, College of Sciences, Princess Nourah bint Abdulrahman University, P.O. Box 84428, Riyadh 11671, Saudi Arabia; 3 Laboratory of Advanced Materials and Interfaces (LIMA), Faculty of Science, University of Monastir, Avenue of Environnment, Monastir 5000, Tunisia; gassoumibouzid2016@gmail.com; 4 Department of Biology, College of Science, Qassim University, Buraydah 51452, Saudi Arabia; 140208@qu.edu.sa; 5 Department of Microbiology, Noakhali Science and Technology University, Noakhali 3814, Bangladesh; noimul.microbiology.nstu@gmail.com; 6 Laboratory of Heterocyclic Chemistry, Natural Products and Reactivity (LR11Es39), Medicinal Chemistry and Natural Products, Faculty of Science, University of Monastir, Avenue of Environment, Monastir 5000, Tunisia; horchani.mabrouk@gmail.com (M.H.); hichem.bjannet@gmail.com (H.B.J.); 7 Quantum and Statistical Physics Laboratory, Faculty of Science, University of Monastir, Monastir 5079, Tunisia; houcineghalla@yahoo.fr; 8 Graduate School of Pharmaceutical Sciences, Nagasaki International University, 2825-7, Huis Tem Bosch, Sasebo, Nagasaki 859-3298, Japan; yfujii@niu.ac.jp; 9 Graduate School of NanoBiosciences, Yokohama City University, 22-2, Seto, Kanazawa-ku, Yokohama 236-0027, Japan; ozeki@yokohama-cu.ac.jp

**Keywords:** thymidine, microorganism, DFT, molecular dynamics simulation, monkeypox, Marburg virus

## Abstract

**Background/Objectives:** Nucleoside precursors and derivatives play pivotal roles in the development of antimicrobial and antiviral therapeutics. The 2022 global outbreak of monkeypox (Mpox) across more than 100 nonendemic countries underscores the urgent need for novel antiviral agents. This study aimed to synthesize and evaluate a series of 5′-*O*-(palmitoyl) derivatives (compounds **2**–**6**), incorporating various aliphatic and aromatic acyl groups, for their potential antimicrobial activities. **Methods:** The structures of the synthesized derivatives were confirmed through physicochemical, elemental, and spectroscopic techniques. In vitro antibacterial efficacy was assessed, including minimum inhibitory concentration (MIC) and minimum bactericidal concentration (MBC) determinations for the most active compounds (**4** and **5**). The antifungal activity was evaluated based on mycelial growth inhibition. Density functional theory (DFT) calculations were employed to investigate the electronic and structural properties, including the global reactivity, frontier molecular orbital (FMO), natural bond orbital (NBO), and molecular electrostatic potential (MEP). Molecular docking studies were conducted against the monkeypox virus and the Marburg virus. The top-performing compounds (**3**, **5**, and **6**) were further evaluated via 200 ns molecular dynamics (MD) simulations. ADMET predictions were performed to assess drug-likeness and pharmacokinetic properties. **Results:** Compounds **4** and **5** demonstrated remarkable antibacterial activity compared with the precursor molecule, while most derivatives inhibited fungal mycelial growth by up to 79%. Structure-activity relationship (SAR) analysis highlighted the enhanced antibacterial/antifungal efficacy with CH_3_(CH_2_)_10_CO– and CH_3_(CH_2_)_12_CO–acyl chains. In silico docking revealed that compounds **3**, **5**, and **6** had higher binding affinities than the other derivatives. MD simulations confirmed the stability of the protein-ligand complexes. ADMET analyses revealed favorable drug-like profiles for all the lead compounds. **Conclusions:** The synthesized compounds **3**, **5**, and **6** exhibit promising antimicrobial and antiviral activities. Supported by both in vitro assays and comprehensive in silico analyses, these derivatives have emerged as potential candidates for the development of novel therapeutics against bacterial, fungal, and viral infections, including monkeypox and Marburg viruses.

## 1. Introduction

**Figure 1:**
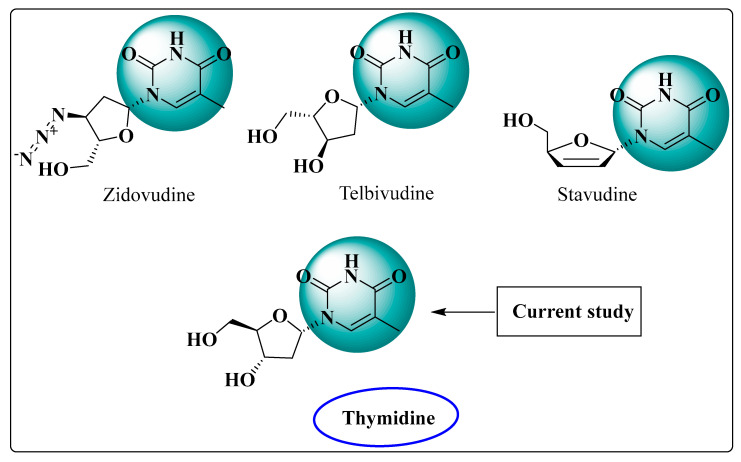
Different derivatives are used as drugs with pyrimidine moieties.

Nucleosides play vital roles in intermediary metabolism, macromolecule biosynthesis, and cell signaling by acting on purinergic receptors [1]. Nucleoside derivatives have been developed artificially and are used as therapeutic drugs. Nucleoside-based drugs function by interfering with viral or cancer cell reproduction, usually by blocking enzymes involved in nucleic acid synthesis. Antiviral nucleoside analogs are used as drugs to treat viral infections by inhibiting the replication of the virus. They work by mimicking the structure of natural nucleosides, which are incorporated into the viral DNA or RNA during replication, thereby disrupting the process. Acyclovir is used to treat herpes simplex virus (HSV) infections [2]. Lamivudine (3TC) is used to treat HIV and hepatitis B virus infections [3]. Anticancer nucleoside analogs, as drugs, interfere with DNA synthesis in rapidly dividing cancer cells, leading to cell death. They may also be used as antimicrobials [4]. The DNA chain is either disrupted or terminated by these molecules, as they bind to the DNA structure during DNA replication. Cytarabine (Ara-C) is administered to patients with acute myeloid leukemia (AML) or acute lymphocytic leukemia (ALL) for treatment [5]. Gemcitabine is utilized in the treatment of pancreatic cancer, non-small cell lung cancer, and certain other solid malignancies [6]. Moreover, it has been employed in antineoplastic chemotherapeutic regimens in conjunction with numerous pyrimidine-directed antimetabolites [7]. Recently, three nucleoside analogs, including 3′-azidodeoxythymidine, which is an antiretroviral medicine, have been used therapeutically for the treatment of acquired immune deficiency syndrome (AIDS) patients [8,9]. In 1966, stavudine was first described, and it is used as a generic medication [10]. It is a type of nucleoside derivative that acts as an inhibitor of HIV-1 reverse transcriptase, and telbivudine is another analog with activity against HBV DNA polymerase [11,12] (Figure 1). In particular, specific forms of carbohydrates and glycans are related to numerous diseases, including cancer, inflammation, and bacterial infections. Different interactions between glycans and lectins have led to advances in immunotherapies, antiviral drugs, and vaccines [13,14].

Antimicrobial resistance (AMR) has emerged as one of the most important threats to global health today [15]. AMR manifests when microorganisms, encompassing bacteria, fungi, parasites, and viruses, undergo evolutionary processes leading to resistance against antimicrobial medications [16,17]. The prevalent issue is largely attributed to the repercussions of antibiotic overuse or misuse across diverse contexts, primarily medical treatment, agricultural practices, animal health care, war crises, and dietary practices [18]. In the absence of preventive interventions, predictions suggest that by 2050, AMR might exceed all other causes of death worldwide [19]. Throughout the world, estimates indicate that there were 1.2 million direct fatalities from AMR in 2019, with an anticipated rise to about 10 million deaths per year by 2050 if inadequate measures to combat AMR are implemented [20].

Currently, the global scientific community increasingly acknowledges that we are entering a post-antibiotic era, marked by the diminishing efficacy of conventional antibiotics against resistant pathogens. This urgent threat necessitates the development of innovative and effective antimicrobial agents to address the growing challenge of antimicrobial resistance [21]. It is believed that tuberculosis is a threatening infection, with an estimated two million mortalities annually [22]. The structural features of nucleosides, in particular, indicate their potential for interacting and interfacing with DNA and RNA and the related enzyme machinery [23]. Nucleosides are formed by combining nitrogenous bases (purine and pyrimidines) with pentose sugars (ribose or deoxyribose) via a *β*-glycosidic bond [24]. In purines and pyrimidines, the *β*-glycosidic bond eliminates a water molecule by joining the sugar C-1′ with the hydrogen atom of *N*-9 or *N*-1 [25]. As a result, pyrimidine nucleosides are *N*-1 glycosides, whereas nucleosides are *N*-9 glycosides. In general, nucleosides are named according to the individual purines or pyrimidines that they contain. Ribonucleosides include ribose, whereas deoxyribose is known as a deoxyribonucleoside. Our study compound is thymidine, which is a nucleoside-based compound termed a pyrimidine deoxynucleoside.

Ribofuranose or deoxyribofuranose of nucleosides can be modified by changing sugar substituents, replacing oxygen with another atom, adding heteroatoms to the sugar ring, changing the ring size, or replacing it with an acyclic moiety [26,27]. These modifications can greatly affect their biological activity and toxicity, contingent upon their physical and chemical attributes [28]. The transformed compounds have a wide range of biological influences [29]. As a result, other methods have been proposed, including direct acylation [30], dibutyltin oxide [31], and enzymatic methods. The direct method produces analogs with 5′-*O*-substituents, whereas the dibutyltin oxide method yields analogs with 3′-*O*-substituents [32]. Most of the time, however, direct methods produce good yields at a relatively low cost [33].

Monkeypox (Mpox) virus is a member of the *Orthopoxvirus* genus. Other human viruses, including the smallpox-causing Variola major virus, Cowpox virus, and Vaccinia virus, are closely related to the virus [34]. Individuals afflicted with human monkeypox often experience frequent occurrences of headaches, fevers, and symptoms such as those of the flu. The characteristic pox scars manifest shortly after the onset of symptoms [35]. The Mpox virus spreads via significant respiratory droplets, salivary secretions, close or direct contact with skin lesions, and possibly through tainted materials or products touched by an infected human being [36]. Mpox vaccine development is challenging for a multitude of reasons. The process of generating a vaccination is both time-consuming and costly [37]. Finding alternatives, such as researching novel medications, appears to be a logical course of action. Consequently, the development of effective Mpox inhibitors may be facilitated by the integration of in silico and in vitro investigations into drug stability and activity.

In recent decades, many studies have been performed in the nucleoside field to obtain different derivatives that have antimicrobial and toxicological activities [38]. Azido groups can be added, and acylation and halogenation can occur in nucleobases to obtain nucleoside derivatives [39,40]. Acylation is crucial for introducing functional groups into molecules, altering their properties and reactivity. Acylation reactions are used to create active compounds and modify the activity of drugs [41]. In the current research, we utilized direct methods to explore the thermodynamics and spectral and biological changes in thymidine. In this work, we provided the results of an in vitro antimicrobial assessment, molecular docking against monkeypox and Marburg viruses with SAR, molecular dynamics analysis, and protein–ligand interactions. Moreover, DFT was applied to predict the global reactivity, FMO, NBO, MEP, pharmacokinetic and drug-likeness properties of the synthesized thymidine derivatives for the first time.

## 2. Results and Discussion

### 2.1. Chemistry

**Scheme 1:**
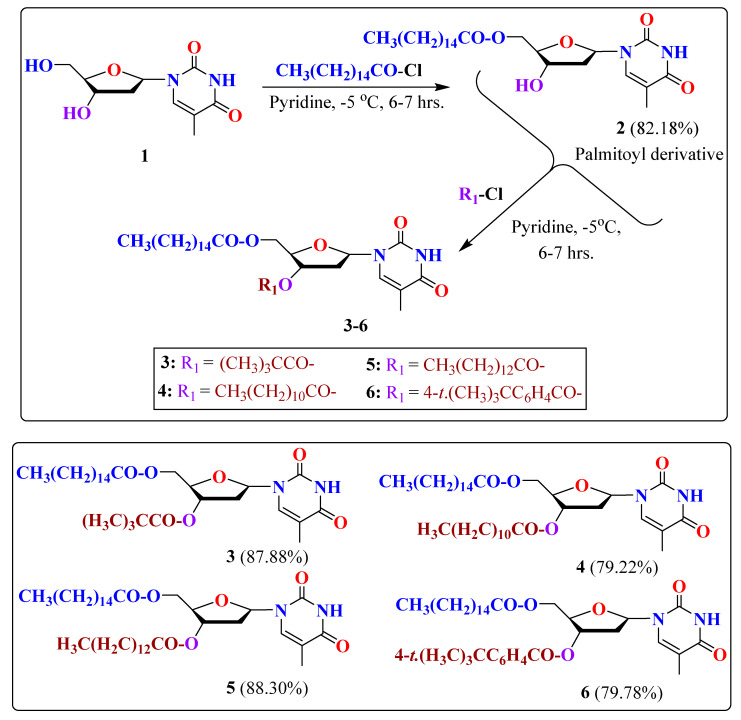
Synthetic pathway. Dry Py, 0 °C, 6 h; DMAP, R_1_-Cl = various acyl halides, 0 °C to rt, stirred for 6−7 h (**2**–**6**).

**Figure 2:**
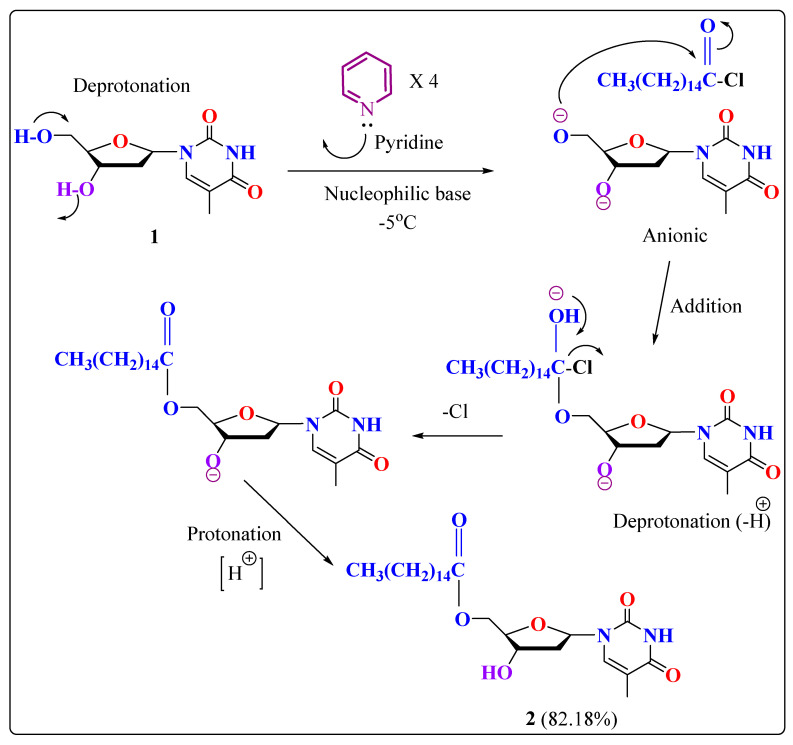
Probable mechanism of the palmitoylation of thymidine.

The main goal of the work was to selectively convert palmitoyl chloride through the direct acylation process (Scheme 1) to the palmitoylated thymidine (**1**). Different aliphatic or aromatic acylating agents have been used to synthesize many chemicals from palmitoylation, and the probable mechanism is illustrated in Figure 2. The selective acylation of a particular hydroxyl group greatly affects nucleoside chemistry. The resulting acylation products could be useful building blocks for the synthesis of new chemical medicines that are more compatible with biological systems [42]. Furthermore, the acyl derivatives illustrated herein could serve as various components involved in the synthesis of numerous other important nucleoside compounds.

### 2.2. Characterization

Our first aim was to obtain thymidine (**1**) to react with a 1:1 molar ratio of palmitoyl chloride in anhydrous pyridine at low temperatures. The palmitoyl derivative (**2**) was subjected to silica gel chromatography, resulting in high yields. In the FTIR spectrum (Figure S1) of compound **2**, the band at 3406–3501 cm^−1^ was attributed to -OH stretching, whereas the band at 1702 cm^−1^ was assigned to -CO (Figure S1). The ^1^H-NMR spectra (Figure S2) revealed a two-proton multiplet at δ 2.32 and δ 1.68, a twenty-six-proton multiplet at δ 1.29, and a three-proton triplet at δ 0.90. This confirmed that the compound had one palmitoyl group. To introduce the 5′-position palmitoyl group, the downfield shift of C-5′ was adjusted to δ 5.0 and 4.92 from its normal δ value (~4.0) [43]. The attachment of the palmitoyl group to the main hydroxyl group of the ribose moiety at the 5′-position, which is the most reactive and least sterically hindered, contributes to the formation of compound **2**. The molecular ion peak at *m*/*z* [M + 1]^+^ 481.60 indicates the chemical formula C_25_H_44_O_5_N_2_CO. The structure of the compound, 5′-*O*-(palmitoyl)thymidine (**2**), is consistent with the results of a comprehensive examination of its FTIR, ^1^H-NMR, ^13^C-NMR, mass, and other properties (Figure S7) [44].

Direct pivaloylation of the palmitoyl derivative (**2**) resulted, and the pivaloyl derivative (**3**) was obtained as a needle. Owing to carbonyl stretching, the FTIR spectrum of this chemical (**3**) is shown in Figure S1, with an absorption band at 1685 cm^−1^. The ^1^H-NMR spectrum (Figure S3) shows a nine-proton singlet at δ 1.32, which is attributable to the methyl protons of the pivaloyl group and indicates that a pivaloyl group has been added to the molecule. Deshielding of C-3′ protons to δ 5.68 (as m) from precursor compound **2** values (δ 4.30) suggested the insertion of a single pivaloyl group at position 3. This compound’s structure was identified as 3′-*O*-pivaloyl-5′-*O*-(palmitoyl)thymidine (**3**) by analyzing all the spectra (Figure S8).

We then treated compound **2** with two fatty acid halides, lauroyl chloride and myristoyl chloride, in anhydrous pyridine at −5 °C, and performed chromatographic purification; we obtained the 3′-*O*-lauroyl derivative (**4**) and 3′-*O*-myristoyl derivative (**5**), respectively. The FTIR spectra (Figure S1) of both compounds exhibited absorption bands corresponding to -CO stretching at 1725 cm^−1^ and 1711 cm^−1^. The ^1^H-NMR spectrum (Figure S4) of **4** exhibited the following peaks: a two-proton multiplet at δ 2.35 and δ 1.64, a sixteen-proton multiplet at δ 1.29, and a three-proton multiplet at δ 0.91, thereby suggesting the introduction of one lauroyl group in compound **4**. The results of the complete analysis of the FTIR, ^1^H-NMR, ^13^C-NMR (Figure S9), and mass spectra were consistent with the structure of the compound, which was assigned as 3′-*O*-lauroyl-5′-*O*-(palmitoyl)thymidine (**4**). Similarly, the structure of compound 193 was ascertained by analyses of its spectral data and assigned as 3′-*O*-myristoyl-5′-*O*-(palmitoyl)thymidine (**5**) (Figures S5 and S10).

Finally, compound **2** was treated with 4-*t*-butylbenzoyl chloride via the same procedure, and the resulting 4-*t*-butylbenzoyl derivative (**6**) was obtained. In its ^1^H-NMR spectrum (Figure S6), two two-proton multiplets at δ 8.05 (Ar-H) and δ 7.51 (Ar-H) and one singlet at δ 1.37 (9H, s, (C*H*_3_)_3_C-) corresponded to the presence of a 4-*t*-butylbenzoyl group in the molecule. The rest of the spectrum was consistent with the structure of the compound assigned as 3′-*O*-(4-*t*-butylbenzoyl)-5′-*O*-(palmitoyl)thymidine (**6**) (Figure S11). These derivatives of the isolated compounds were synthesized through the use of a wide range of rarely used acylating agents to facilitate structural elucidation and provide new synthetically important compounds.

### 2.3. Antibacterial Potential

Nucleoside derivatives represent a promising class of novel antimicrobial agents due to their predicted genomic-level mechanisms of action, which are anticipated to disrupt essential microbial transcription or replication processes. Given the absence of alternative metabolic pathways in many pathogenic microorganisms, nucleoside derivatives exhibit high potential as targeted antimicrobial therapeutics [44]. Antibacterial activity was assessed via a disc diffusion assay, and the results are summarized in Table 1 and Figure S12. Compound **4** demonstrated the most potent inhibitory effect against *Escherichia coli*, yielding a zone of inhibition of 20.75 ± 0.2 mm, surpassing that of the reference antibiotic azithromycin (17.25 ± 0.1 mm). Furthermore, compound **4** exhibited significant efficacy against *Bacillus cereus* (17.00 ± 0.4 mm) and *Pseudomonas aeruginosa* (17.75 ± 0.2 mm). Compound **5** also displayed notable antimicrobial activity, with inhibition zones of 15.00 ± 0.2 mm for *B. cereus* and 14.00 ± 0.1 mm for *P. aeruginosa*. Additionally, compounds **2** (7.84 ± 0.1 mm), **3** (7.75 ± 0.2 mm), and **5** (9.26 ± 0.2 mm) exhibited measurable inhibitory effects on the Gram-negative bacterium *E. coli* (12.75 mm). On the basis of these findings, the order of antibacterial potency among the thymidine derivatives was determined to be **4** > **5** > **2** > **3** > **6** on the basis of the aforementioned observations. Numerous studies documented in the scientific literature have demonstrated that certain pyrimidine nucleoside derivatives possess significant antimicrobial potential. The antimicrobial efficacy of these derivatives, which are functionalized with various acyl moieties, was assessed in vitro via disc diffusion and food poisoning techniques against a range of human and plant pathogenic microorganisms. The results revealed that the synthesized compounds exhibited moderate to strong antibacterial and antifungal activities [7]. Several marine-derived nucleosides, such as pyrimidine-1-β-D-ribosides, have demonstrated selective antibacterial activity, particularly against *Staphylococcus epidermidis* [45]. Several novel thiopyrimidine nucleoside analogs, particularly the blocked cyclic nucleosides derived from thieno [2,3-d]pyrimidine scaffolds, have also been reported to exhibit notable antimicrobial activity against *Escherichia coli*, *Bacillus subtilis*, *Aspergillus niger*, and *Candida albicans*, as evaluated by the disc diffusion method [46].

### 2.4. MIC and MBC Evaluation

**Figure 3:**
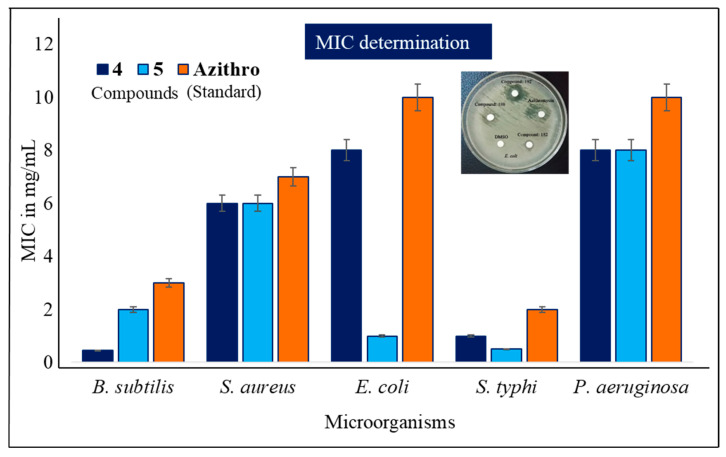
MIC values of the compounds against pathogens. The data are presented as mean ± SD, and the values are represented for triplicate experiments. Statistically significant inhibition (*p* < 0.05) for the test compounds and reference antibiotic azithromycin.

**Figure 4:**
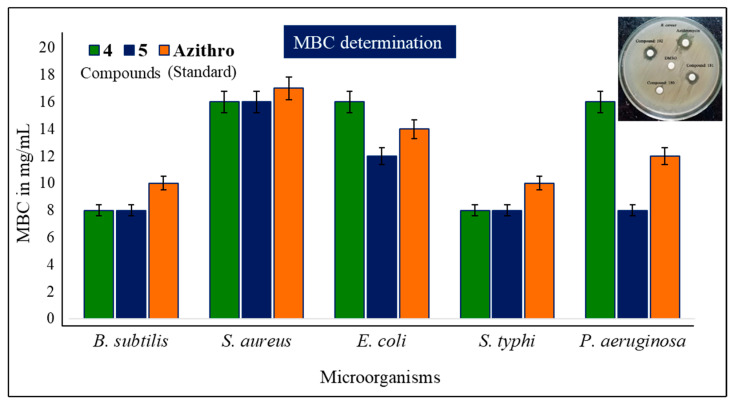
MBC values of the compounds against pathogens. The data are presented as mean ± SD, and the values are represented for triplicate experiments. Statistically significant inhibition (*p* < 0.05) for the test compounds and reference antibiotic azithromycin.

Furthermore, the minimum inhibitory concentration (MIC) and minimum bactericidal concentration (MBC) of the most potent thymidine derivatives were determined to assess their bactericidal efficacy. The detailed results are presented in Tables S2 and S3, and Figure 3 and Figure 4. Among the tested compounds, derivatives **4** and **5** demonstrated the most pronounced antibacterial activity, with MIC values ranging from 0.45 to 8.0 µg/mL. Compound **4** exhibited broad-spectrum efficacy, showing potent activity against all the bacterial strains tested, with the lowest MIC of 0.45 µg/mL recorded against *Bacillus subtilis*. Notably, compound **5** also displayed significant antibacterial activity against multiple pathogens, with a particularly strong effect against *Salmonella typhi* at concentrations as low as 0.5 µg/mL.

Compounds **4** and **5** presented the MBC values, at 8.00 µg/mL, against *Bacillus subtilis* and *Salmonella typhi*. In contrast, the highest MBC values for these compounds were observed against *Escherichia coli*, *Staphylococcus aureus*, and *Pseudomonas aeruginosa*, reaching 16.00 µg/mL. We can assume that thymidine derivatives **4**/**5** disrupt bacterial cell wall synthesis (MIC 0.45–8 µg/mL; MBC 8–16 µg/mL) via peptidoglycan transpeptidase/lipid II targeting, overcoming resistance-linked efflux/biofilms in Gram± pathogens and demonstrating broad-spectrum bactericidal efficacy. Overall, the MBC values for both compounds ranged from 8.00 to 16.00 µg/mL, underscoring their bactericidal potential against a broad spectrum of pathogenic organisms [47]. Nucleoside-based antimicrobial agents disrupt key cellular functions in bacterial and fungal pathogens, including nucleoside metabolism, cell wall formation, and the synthesis of nucleic acids and proteins. Some nucleoside analogs are also known to influence additional metabolic pathways, although their precise mechanisms have not yet been fully elucidated. These unexplored interactions offer potential for discovering new antimicrobial drugs with distinct mechanisms of action [48].

### 2.5. Antifungal Susceptibility

As presented in Table 2 and Figure S13, several thymidine derivatives strongly inhibited the mycelial growth of both *Aspergillus niger* and *Aspergillus flavus*. Among the tested compounds, compound **2** exhibited notable antifungal activity, as it inhibited *A. niger* and *A. flavus* by 73.72 ± 1.2% and 77.45 ± 1.0%, respectively. Compound **3** significantly inhibited *A. niger* (77.54 ± 1.1%) but had no inhibitory effect on *A. flavus*. In the mycelial growth assay, compound **4** effectively suppressed the growth of *A. niger* and *A. flavus*, with inhibition rates of 64.40 ± 1.3% and 74.59 ± 1.2%, respectively. Compound **5** also exhibited considerable antifungal activity, reducing the mycelial proliferation of *A. niger* and *A. flavus* by 55.61 ± 1.1% and 61.52 ± 1.0%, respectively. In general, thymidine derivatives likely exert antifungal effects through multiple mechanisms, including cell wall destabilization, membrane disruption, and intracellular metabolic interference.

The antifungal activities of compounds **2**, **3**, **4**, and **5** against *A. niger* were generally more pronounced than their activities against *A. flavus*. Notably, the inhibition zones produced by these derivatives surpassed those of the reference antifungal drug nystatin, as indicated in Table 2. In contrast, compound **6** and the parent molecule thymidine (compound **1**) had no inhibitory effects on either fungal strain. Previous studies have shown that structural modification of thymidine, particularly through acylation, enhances its antimicrobial properties. It has been reported that certain derivatives of uridine and thymidine, functionalized with triethylamine or trimethylamine groups, exhibit concentration-dependent antifungal activity in vitro against two *Candida* species [49]. Certain promising nucleosides, particularly *N*^4^-modified cytidines, exhibit potent inhibitory activity against filamentous fungi known to cause biodeterioration of cultural heritage materials, such as tempera paintings. These compounds demonstrate antifungal efficacy at sublethal concentrations ranging from 0.1 to 0.5 mM, whereas complete growth inhibition occurs at lethal doses between 0.5 and 3 mM [50].

### 2.6. SAR Assessment

The development of antimicrobial agents remains an urgent and dynamic area of research driven by the increasing prevalence of multidrug-resistant bacteria and the emergence of novel infectious pathogens. Many antimicrobial compounds are built upon five-membered heterocyclic frameworks, which are fundamental to biological activity across a wide range of organisms [51]. In particular, fused or condensed ring systems have garnered significant interest because of their versatility as pharmacophores and their broad spectrum of physiological effects [52]. Among these, nucleoside derivatives represent a logical and highly promising class of antimicrobial agents owing to their ability to target key enzymatic processes involved in the biosynthesis of critical cellular components such as peptidoglycans in bacteria, chitins in fungi, and proteins.

**Figure 5:**
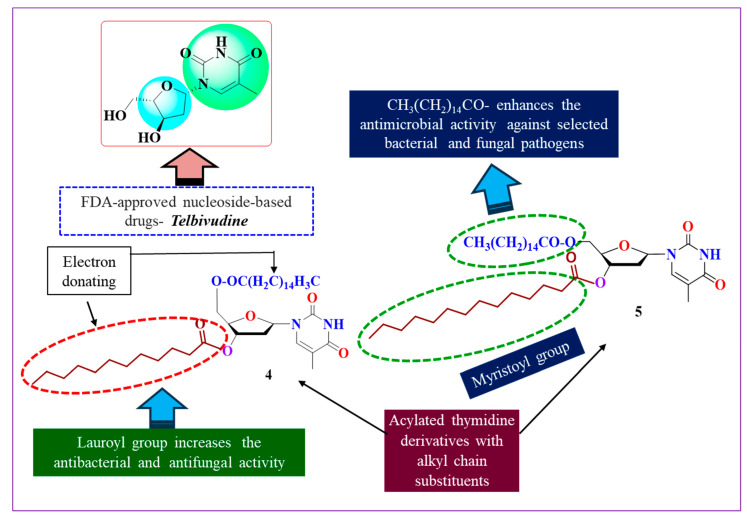
Structure–activity studies of thymidine derivatives.

Because nucleosides play essential roles in cellular metabolism, modifying their structure can yield compounds with potent bioactivity. As illustrated in Figure 5, structure–activity relationship (SAR) analysis provides valuable insights into how thymidine-based molecules exert their antimicrobial effects [53]. Data from antibacterial assays (Table 1 and Table 2) reveal that structural modification of the thymidine scaffold significantly enhances its antimicrobial potency, as the unmodified parent molecule lacks intrinsic activity. Among the tested acylated derivatives, the following order of increasing antibacterial efficacy was observed for both Gram-positive and Gram-negative strains: lauroyl > myristoyl > palmitoyl > pivaloyl > 4-tert-butylbenzoyl. As a result, the following became evident:

✓ The potential implications for long-chain aliphatic hydrocarbons (lauroyl, myristoyl);
✓ The binding acquaintances are enhanced by the attachment of the palmitoyl-substituted acyl group at position 5′;
✓ By combining the acyl groups that are substituted at position 3′ with the lauroyl and myristoyl groups, the binding affinity is increased;
✓ The presence of pyrimidine and ribose rings is also important for the efficacy of the activity.

Compounds **4** and **5**, bearing lauroyl and myristoyl groups, respectively, displayed the most potent activities across the tested microorganisms, confirming the importance of acyl chain length and hydrophobicity in modulating bioactivity. These modified thymidine compounds affect fungal cells at multiple levels [53]. Their long fatty acid chains, like molecular anchors, burrow into the cell membrane, destabilizing its structure and causing leakage. The palmitoyl group, which is attached at a key position, locks the compound firmly in place, whereas bulkier side chains (such as myristoyl) jam critical enzymes. Moreover, the core structure, the pyrimidine and ribose rings, acts as a Trojan horse, mimicking natural building blocks and disrupting DNA or protein synthesis. Together, these mechanisms influence fungal growth, which explains why some derivatives outperform even standard antifungal drugs. The fungi are structurally obstructed from the inside across, not only suppressed.

Compared with their palmitoyl analogs, lauroyl- and myristoyl-substituted thymidine derivatives exhibited higher antibacterial activity, whereas pivaloyl and 4-tert-butylbenzoyl moieties demonstrated comparatively weaker effects. In this study, higher concentrations of synthetic compounds (ranging from 0.45 to 8.0 µg/mL) were required to effectively inhibit the growth of Gram-negative bacteria. This disparity is attributed to fundamental differences in bacterial cell wall architecture. The outer membrane of Gram-negative bacteria, enriched with lipopolysaccharides (LPSs), serves as a permeability barrier that restricts the diffusion of antimicrobial agents and impedes peptidoglycan-targeted transport mechanisms [54]. Moreover, the periplasmic space of Gram-negative bacteria contains hydrolytic enzymes capable of degrading xenobiotic compounds, further contributing to resistance [55].

**Figure 6:**
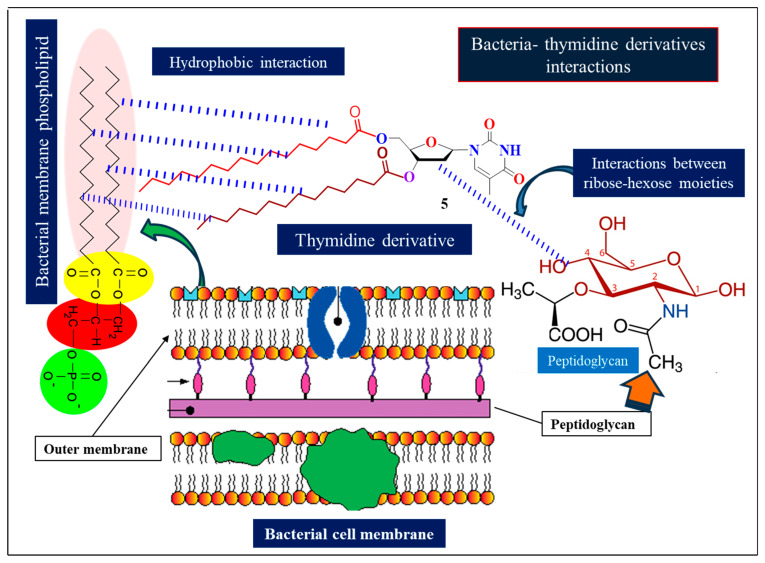
Membrane permeability with hydrophobic interactions between the bacterial outer membrane and peptidoglycan with compound **5**.

Gram-positive bacteria, on the other hand, have a thick, porous peptidoglycan layer that allows lipophilic molecules to pass through even if they lack an outer membrane [56]. Gram-positive bacteria were therefore often more vulnerable to the thymidine derivatives that were examined. The derivatives became more hydrophobic as the lauroyl and myristoyl concentrations increased. This physicochemical characteristic is strongly associated with increased membrane permeability, biological activity, cytotoxicity, and membrane integrity disturbance. Judge’s [57] previous investigations highlighted the significance of hydrophobic interactions between lipid bilayers and alkyl chains in regulating the antibacterial efficacy of aliphatic alcohols through lipid solubility. Hydrophobic interactions between cylindrical thymidine derivatives and lipid-rich bacterial membranes are likely to compromise membrane integrity and ultimately result in bacterial cell death (Figure 6).

### 2.7. Optimized Structures and MEP

**Figure 7:**
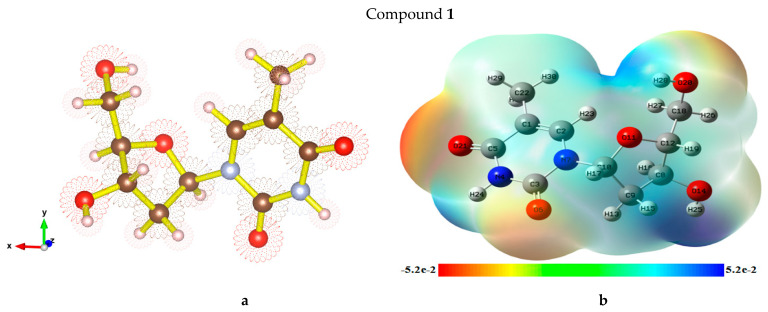

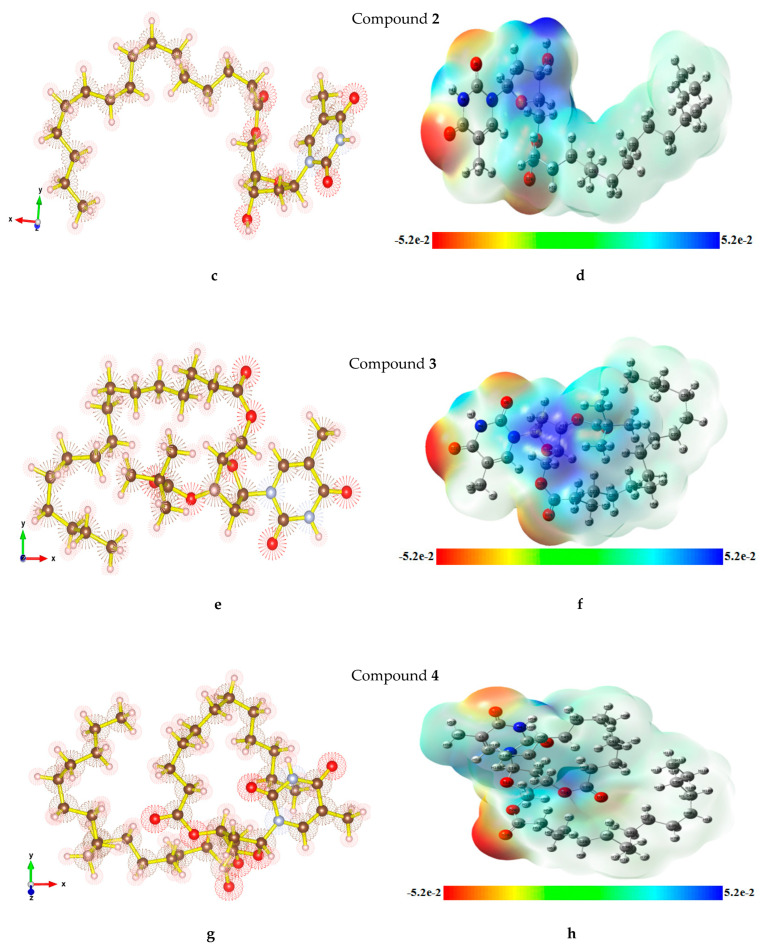

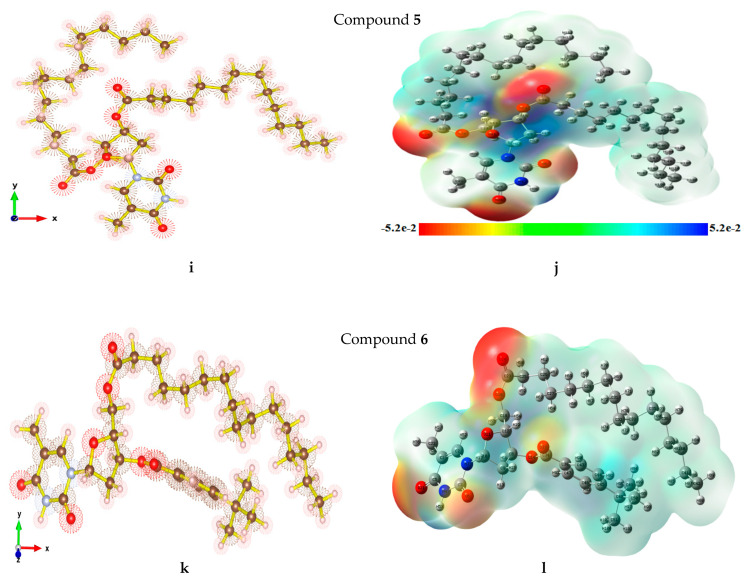
Optimized structures and MEPs of all the studied systems (**1**–**6**). (**a**,**c**,**e**,**g**,**i**,**k**) indicate the active sites of the compounds, and (**b**,**d**,**f**,**h**,**j**,**l**) indicates the MEP of the compounds.

The molecular electrostatic potential (MEP) is a powerful tool for understanding interactions, chemical reactivity, and repartitions of active sites on the surface of a studied compound [58,59]. In the MEP surface area, the red color region (acceptor groups) indicates the excess accumulation of electrons in each group, the blue color (donating groups) indicates poorly electronic regions, and the green-yellow color indicates that this region is neutral. The 3D-optimized structure and MEP iso-surface of all the studied compounds (**1**–**6**) are depicted in Figure 7. Compounds **1**–**3** have three active sites around the oxygen atoms. These regions are electron acceptors, so it is possible to form strong hydrogen-type interactions with the target protein. These findings indicate that these ligands may be effective in forming a set of strong interactions with the chosen protein, thereby inhibiting its activity. In addition, from the 3D-MEP data in Figure 7b,d,f, we deduced that the ligands present negative electronic accumulation at the end and positively charged sites located in the center. These results demonstrate that electronic charge transfer occurs on the surface of the ligands (**1**–**3**). Let us deduce the good stability of these ligands. Additionally, the excess free electrons are good indicators of the formation of a donor-acceptor bond with the selected protein. Hence, these ligands are effective for such biological applications. Moreover, concerning compound **4**, there are two acceptor sites (deep red) behind the C5-O21 and C3-O2 groups. These two regions are possible sites of attack for the protein. They are capable of fixing proteins through strong hydrogen bonding interactions. The other sites are practically electronically poor (light blue); all the free electrons are grouped around the oxygen atoms (Figure 7h). This finding demonstrates that this ligand may not be very effective against a particular protein. The existence of an excess of free electrons on the surface of our systems is an advantage in facilitating the formation of an electron donor–acceptor coupled with the protein and hence the fixation and stability of the ligand–receptor complex. Compared with the other systems, compounds **5** and **6** are more electronically charged; they have four active sites (deep red color), three located at the end, and one in the center (Figure 7j,l). This result clearly shows that these two ligands are easily attacked by a significant number of strong hydrogen bonding interactions on the protein, explaining the effectiveness of the inhibition of these proteins by ligands **5** and **6**. This finding is also obtained by docking sections. Ultimately, the 3D-MEP results revealed that compounds **5** and **6** may be very effective against selected proteins.

### 2.8. Frontier Molecular Orbital (FMO)

**Figure 8:**
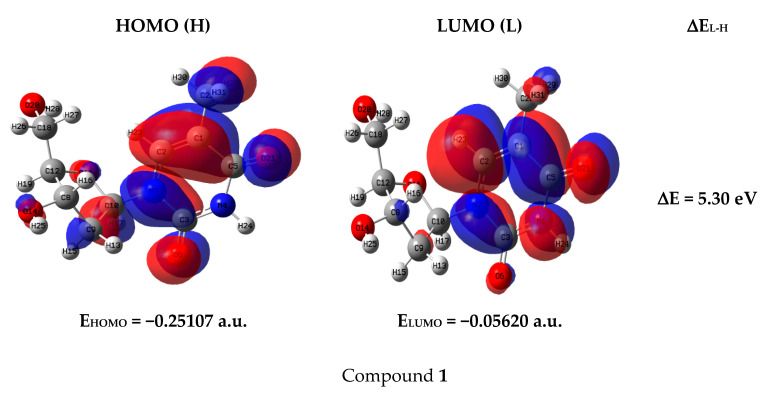

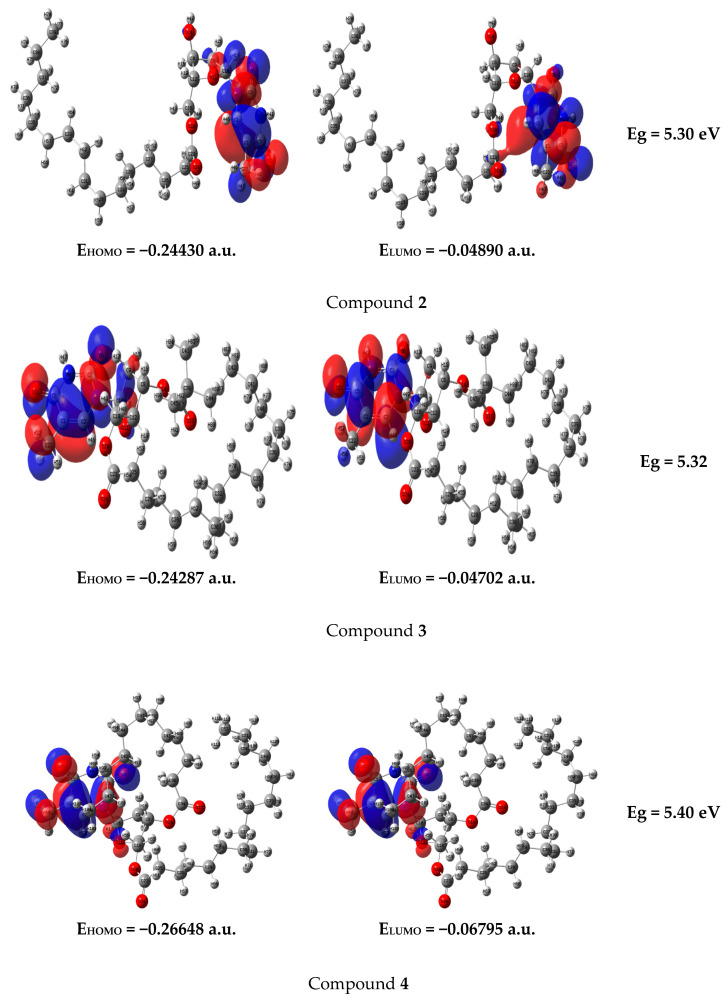

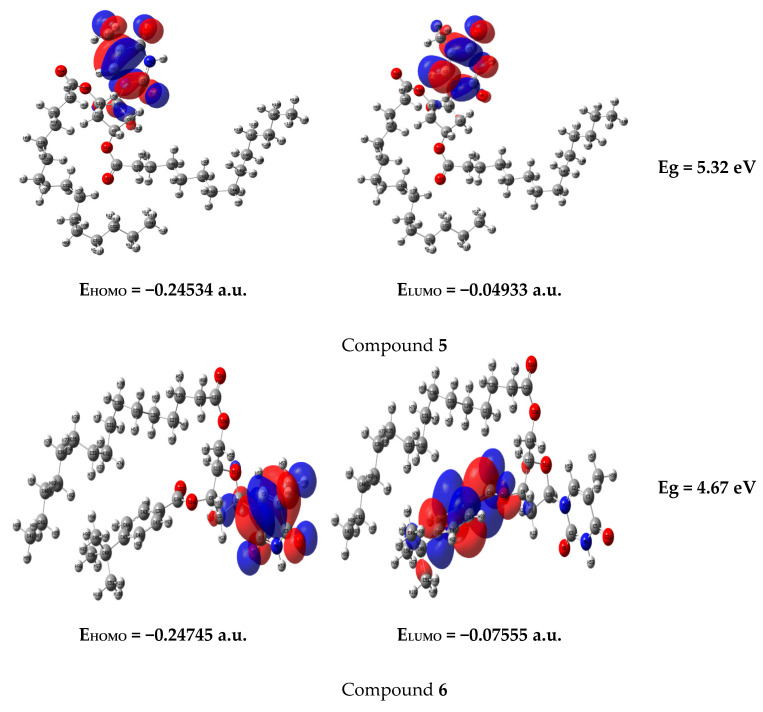
HOMO-LUMO is the surface of all the studied compounds (**1**–**6**) calculated at the B3LYP/6-311G+(d,p) level of theory.

The highest occupied molecular orbital (HOMO) and lowest unoccupied molecular orbital (LUMO) molecular orbitals are the main parameters for determining the electronic locations and distinguishing the active sites on the surface of a material. Additionally, the HOMO and LUMO iso-surfaces describe the ionization potential and electronic affinity of each system [60]. The ability of a molecule to donate or receive electrons is explained by the values of the HOMO and LUMO energies. Additionally, the stability and electron-donating or electron-accepting nature of a molecule are obtained with the HOMO-LUMO (Δ_EL-H_) gap energy [61]. The 3D plots of the frontier molecular orbitals in all the compounds (**1**–**6**) are depicted in Figure 8. The ΔE_L-H_ gap energy is calculated for each compound in Figure 8. The HOMO and LUMO molecular orbitals in compound **1** are localized on the group surface, which contains three oxygen atoms. The gap energy of compound **1** is 5.30 eV. For compounds **2**, **3**, and **5**, there was no clear change in the location of the molecular orbitals (HOMO and LUMO) in comparison with that of molecule **1**. This idea suggests that perhaps the electrons are quite fixed by fairly strong electrostatic forces in this active zone, which coexist with the accumulation of a significant number of acceptor oxygen atoms. These results were verified utilizing MEP analysis. The added ramifications appear poor electronically, so it may act as a barrier that leaves free electrons trapped in its active site. The excess electronic accumulation in this zone is very beneficial for protein attack. This result is a good idea because our compounds maintain electronic charge transfer (donor-acceptor coupling) with the guest. Therefore, there is a possibility of the formation of a significant number of hydrogen binding interactions with the target protein. In addition, the presence of oxygen electronegative acceptor atoms improves the presence of free electron acceptors on the surface of materials. This finding will be further explained in the molecular docking section. For compounds **2**, **3**, and **5**, the ΔE_L-H_ peak at approximately 5.3 eV was obtained. In addition, compound **4** presents the highest gap energy, equal to 5.40 eV. The E_HOMO_ and E_LUMO_ values of compound **4** are equal to −0.26648 a.u. and −0.06795 a.u. These values indicate that this compound has a low binding affinity for the target virus. Additionally, this compound requires a low electronic charge transfer to occur on the surface and is less stable than other systems. Figure 8 shows that the HOMO orbitals of compound **6** are located along the central group, after which these electrons migrate to the excited state and are placed surrounding the ligand, which contains two oxygen atoms and two nitrogen atoms. This compound has a lower gap energy, which is 4.67 eV. The E_HOMO_ and E_LUMO_ values of compound **6** are −0.24745 a.u. and −0.07555 a.u., respectively. Therefore, this compound has a high binding affinity and is efficient against the target virus. Additionally, this idea confirmed that high energy/energy transfer occurs on the surface of this material. Moreover, the possibility of the formation of many hydrogen bonding interactions with the selected protein increases the effective potential of this new compound against the selected virus. This result will be discussed in detail in the molecular docking section. Frontier molecular orbital analyses demonstrated that compound **6** may have efficient antiviral activity.

### 2.9. Global Reactivity Parameters

Global reactivity parameters such as electronegativity (χ), hardness (η), softness (S), chemical potential (μ), and electrophilicity (ω) are key descriptors for understanding the stability of a molecule, the donor or acceptor systems, the degree of electronic charge transfer on the surface, and the chemical reactivity of the studied compound [62]. These global reactivity parameters are calculated in terms of the frontier molecular orbital energies as follows:

(1) $$\chi =-\frac{1}{2}(E_{HOMO}+E_{LUMO})$$

(2) $$\eta =-\frac{1}{2}(E_{HOMO}-E_{LUMO})$$

(3) $$S=\frac{1}{\eta }$$

(4) $$\mu =\frac{1}{2}(E_{HOMO}+E_{LUMO})$$

(5) $$\omega =\frac{\chi^{2}}{2\eta }$$

From Table 3, a low value of hardness (η) is obtained for compound **6**, with a value of approximately 2.34 eV. This finding indicates that a high energy/charge transfer occurs on the surface of the material. Therefore, the possibility of forming a donor-acceptor pair of electrons with the selected guest increases the stability of the complex with H-bonding interactions. This idea is very advantageous for the antiviral activity of the studied system. This result is also confirmed by MEP and FMO discussions. In other words, this system is more polarizable than the other systems. The other systems have hardness values varying from 2.66 eV to 2.70 eV. Moreover, compound **4** has the highest value of η at 2.70 eV, demonstrating low electronic energy/charge transfer on the surface. The softness values of compounds **1**–**5** are identical, equal to 0.37 eV. A higher value is obtained for compound **6**, with S equal to 0.42 eV, indicating the good reactivity of this system. This compound is more reactive than others. In addition, it has a high value of electrophilicity (ω) at approximately 4.11 eV. The other systems have ω values less than 4 eV. These findings led to the conclusion that compound **6** has an electrophilic characteristic. This signifies the good acceptance of electrons by this system, indicating that our system has a good capacity to attack a target protein. This idea is determined by MEP/FMO analyses. The lower electrophilicity values of systems **1**–**5** specify the maximal flow of electrons from the donor groups to the acceptor groups containing oxygen atoms. This result confirmed the good stability of these systems.

### 2.10. 3D-NBO Charge

**Figure 9:**
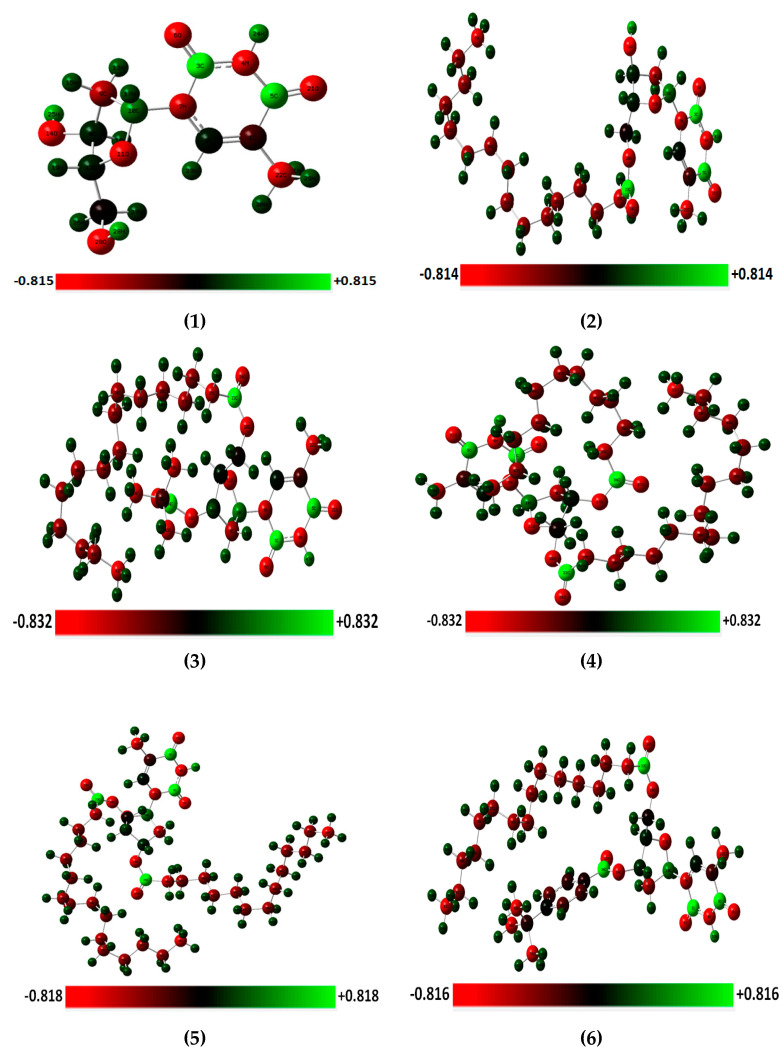
NBO charges of all the studied compounds (**1**–**6**).

To understand the accumulation and electronic distribution on the surface of each compound, we performed an NBO analysis [63]. The 3D-NBO figures are depicted in Figure 9. Compounds **1**–**4** have negatively charged groups, namely, -C-O and -N-C=O. This result indicates that the acceptor sites are located around the oxygen atoms. These groups may be responsible for attacking the targeted protein and forming highly hydrogen bonded interactions with the guest. Additionally, these groups undergo electronic exchange with the protein, which is beneficial for the stability of the ligand–protein complex. In addition, for compounds **5** and **6**, all the groups connected to oxygen atoms present a negative charge, indicating that these systems are more highly charged than the others. Therefore, compounds **5** and **6** may form a greater number of hydrogen bonding interactions with the selected protein than the other compounds do, confirming the antiviral capacity of these systems. This finding will be explained in detail in the molecular docking section. Ultimately, the NBO charges led to the conclusion that compounds **5** and **6** may be very effective against selected viruses.

### 2.11. Molecular Docking Pose and Interaction Analysis of the Monkeypox Virus

**Figure 10:**
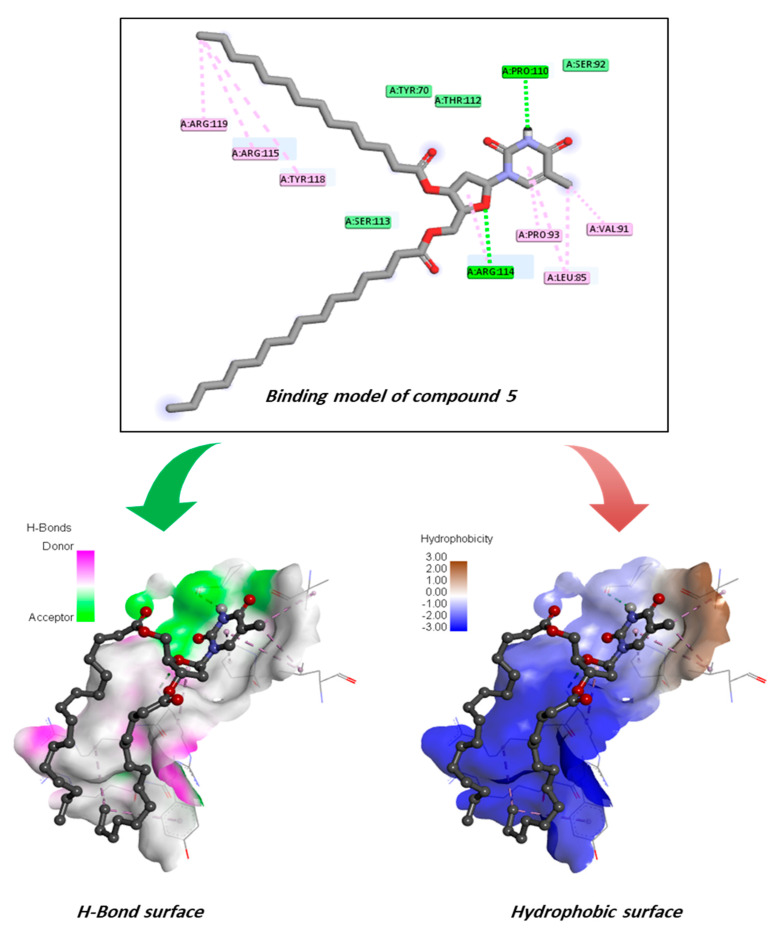
Binding mode of the most effective compound **5** in the binding cavity of the A42R profilin-like protein from the monkeypox virus.

**Figure 11:**
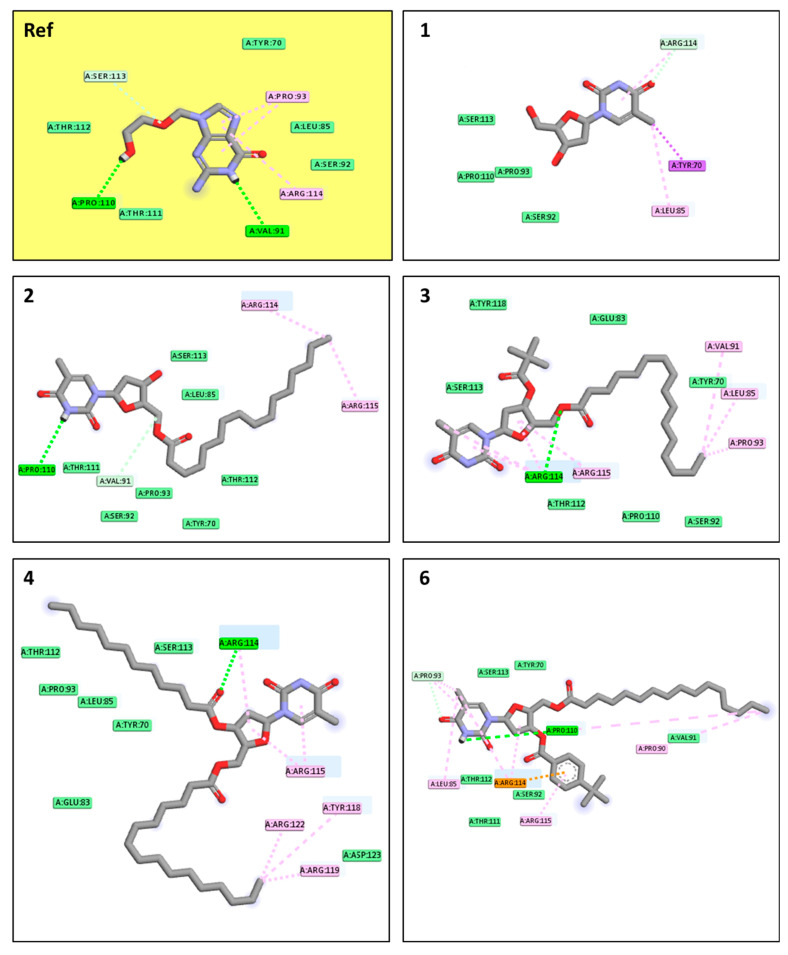
Binding modes of docked compounds **1**, **2**, **3**, **4,** and **6** and the reference (acyclovir) in the binding cavity of the A42R profilin-like protein from the monkeypox virus.

Molecular docking pose and interaction analyses were carried out to evaluate the binding region of the drug-protein complex and the active site after the formation of the docking complex. This part of the investigation included docking interactions between the newly synthesized compounds and the monkeypox virus and their binding affinities. The modeling results revealed that all the ligands bound well within the catalytic pocket of the target receptor, with free binding energies ranging from −6.2 to −4.5 kcal/mol. This significance is proven by these values, which exceed those of the standard docked ligand (acyclovir). Table 4 shows that compound **5** displayed the best docking score (−6.2 kcal/mol) in comparison with its derivatives. Indeed, the results of the in silico experiments reveal potential profound interactions by the docked ligand **5**, in addition to some van der Waals contacts, two conventional hydrogen bonds: a first by the NH group of its ‘2,3-dihydropyrimidin-4(1H)-one’ pharmacophore with Pro110 and a second with Arg114 via its ‘tetrahydrofuran’ moiety. Thus, the importance of H-bond formation in the inhibition of a target protein and in enhancing complex stability (Figure 10) was supported by other contacts as well as the alkyl and pi-alkyl interactions displayed by the **5**’s alkylated chains, which was demonstrated by the hydrophobic surface (Figure 10). These hydrophobic interactions were formed with the following series of amino acids: Leu85, Val91, Pro93, Arg115, Tyr118, and Arg119. As shown in Figure 11, starting material **1** did not display good docking results, confirming the importance of the chosen synthetic route to obtain target products **3**–**6**. For more details, concerning the second effective antiviral ligand **6** (−5.7 kcal/mol), according to the modeling results, its effectiveness is perceptible through the formation of an H-bond with Pro110, in addition to a Pi-Cation with Arg114 and some hydrophobic interactions, whereas ligands **3** (−5.1 kcal/mol) and **4** (−5.0 kcal/mol) are involved in an H-bond with Arg114. For compound **2** (−4.6 kcal/mol), as shown in Figure 11, its predictive antiviral potential is perceptible from an H-bond with Pro110, in addition to a few other contacts.

### 2.12. Molecular Docking Pose and Interaction Analysis of the Marburg Virus

**Figure 12:**
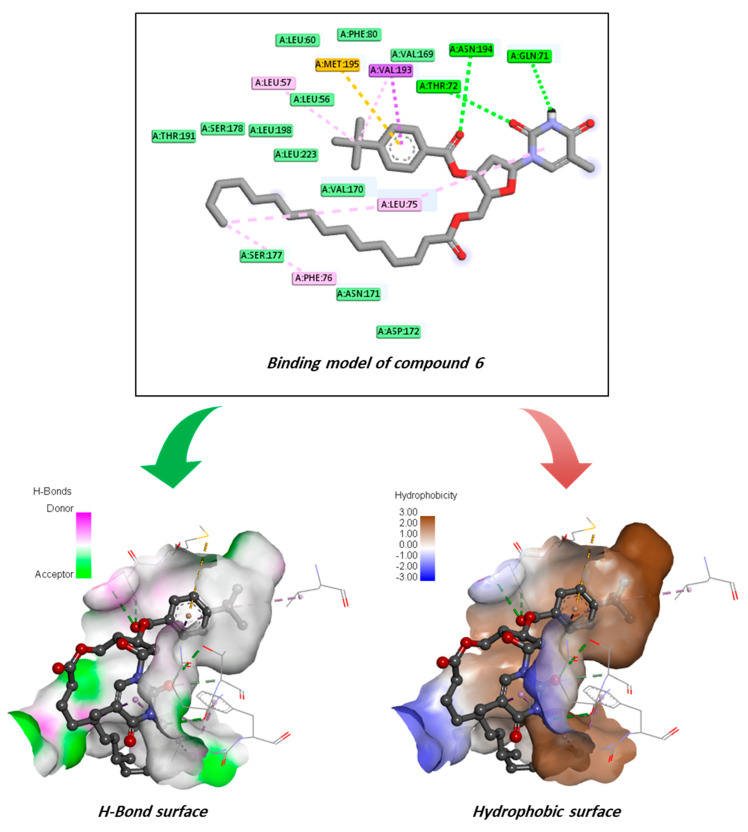
Binding mode of the most effective compound, compound **6**, in the binding cavity of the Marburg virus.

**Figure 13:**
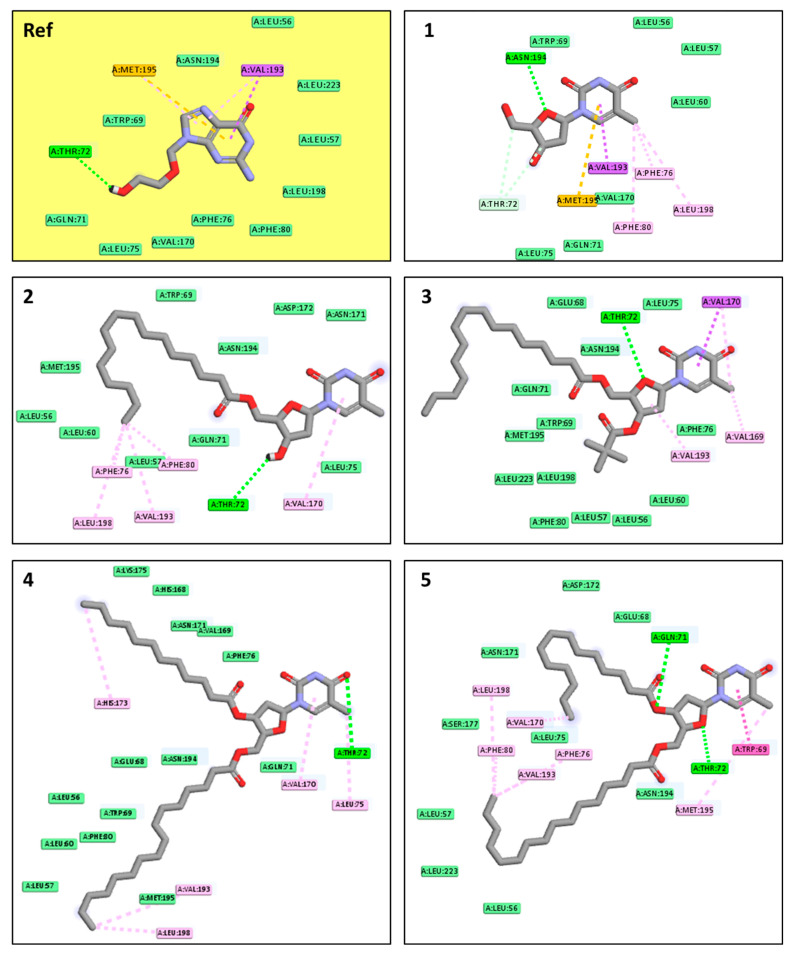
Binding modes of docked compounds **1**, **2**, **3**, **4,** and **5** and the reference (acyclovir) in the binding cavity of the Marburg virus.

To better explore the capacity of the newly synthesized compounds to target Marburg virus, we investigated their binding modes. As the tabulated results show, compound **6**, which has the lowest docking affinity (−7.0 kcal/mol), was found to be the most effective molecule compared with the other docked compounds involving the standard (acyclovir) (Table 5). Notably, ligand **6** could nicely insert into the binding groove of the target receptor (Figure 12), H-bond, and hydrophobic surfaces, forming multiple interactions via three H-bonds through its NH and CO groups with Gln71, Thr72, and Asn194. Moreover, hydrophobic interactions could be observed between the docked ligand and the amino acid residues Leu57, Leu75, Phe76, and Val193. This last residue is involved in a Pi-Sigma interaction with the phenyl ring. This last fragment formed, in turn, a Pi-sulfur contact with Met195. Furthermore, ligand **6** displayed some van der Waals interactions. Therefore, the phenomenon observed might provide a rational explanation for the highly selective Marburg virus inhibition of derivative **6**. On the other hand, compound **5** (−5.6 kcal/mol) was found to be the second most effective ligand, exhibiting two H-bonds formed between the oxygen atoms and Gln71 and Thr72, in addition to some other interesting contacts, such as alkyl interactions with the series of residues Phe76, Phe80, Val170, Val193, and Leu198, indicating the importance of the linked carbon skeleton at the starting synthon 1 during the synthesis reaction, whereas the other docked ligands showed only H-bonds in addition to some other interactions detailed in Figure 13. Therefore, according to the above findings, all these newly synthesized compounds may be possible therapeutic approaches as inhibitors of monkeypox and Marburg virus.

### 2.13. ADMET Prediction

Pharmacokinetics studies drug entry, metabolism, and elimination. The biological and pharmacological features of a drug at its site of action determine how a person reacts to it. This study examined the bioavailability and efficacy of oral chemical medicines [64,65]. Encouraged by this information, ADMET predictions were performed, and the results are shown in Table 6. Indeed, solubility is a crucial criterion for the absorption and distribution of drugs because it has a direct effect on oral bioavailability. It is measured by logS, which can vary between −6 and 0. The closer logS is to 0, the more soluble the molecule is. In our case, compounds **3** and **6** showed interesting solubilities. Caco-2 cell lines, which are human epithelial colorectal adenocarcinoma cells, are cell monolayers that are often used as models of the human intestinal mucosa in vitro to predict the oral absorption of drugs [66,67]. All the tested molecules showed interesting Caco-2 permeability values as well as high intestinal absorption (>30%), suggesting that they would be absorbed in the small intestine, especially compound **4**, whose value was 77.11%. On the other hand, it is a measure of the ability of a substance to cross the skin barrier and enter the bloodstream. In general, a compound is considered to have good skin permeability if its log Kp is less than −2.5. This is the case for all our products tested, as shown in the tabulated data. All the parameters discussed above explain the ‘Absorption Power’. Now moving on to the ‘Distribution’ that manifests from its settings as the VDss (human) (volume of distribution), the circulation in blood plasma is acceptable for compounds with values higher than −0.15. This is the case for compounds **3** and **6**, with log L/kg = 0.011. The fraction unbound, BBB permeability, and CNS permeability values of the tested compounds displayed interesting profiles. With respect to the ‘metabolic’ settings of the target molecules tested, most of these compounds cannot inhibit the main cytochrome (CYP) enzymes: CYP1A2, CYP2C19, CYP2C9, CYP2D6, and CYP3A4. These enzymes are crucial to xenobiotic metabolism in the human body, particularly oxidation, and their inhibition can cause metabolism-related drug–drug interactions, which usually involve competing for the enzyme’s binding active site with another drug. Enzyme inhibition slows the metabolism and clearance of other coadministered xenobiotics, increasing their plasma levels and altering their therapeutic impact. CYP enzyme inhibition can degrade and cause xenobiotic toxicity or reduce a drug’s therapeutic advantages [68]

These findings affirm the better effectiveness of the tested molecules. The fourth factor, ‘Excretion’, was estimated by calculating the renal OCT2 substrate parameter, and all the tested candidates exhibited no renal OCT2 substrate. Finally, we are interested in toxicity, which has crucial value. Indeed, the AMES toxicity test is a widely used strategy for determining the mutagenesis potency of a substance using bacteria. Therefore, a positive test demonstrated that the chemical is mutagenic and consequently has the potential to cause cancer [69]. In this case, compounds **2**, **4**, and **5** have no mutagenesis potential. The maximum tolerated dose (MTD) represents an approximation of a chemical compound’s hazardous dosage threshold in humans. Compound **2** has the lowest MTD (−0.2), whereas compounds **3** and **6** have elevated MTDs (0.438) compared with each other. The hepatotoxicity results revealed that all the ligands except **3** and **6** were hepatotoxic. Concerning the hERG I and II inhibitor capabilities, none of the tested compounds exhibited any effect.

### 2.14. MD Trajectory Data Analysis of Monkeypox (Mpox) Virus

#### 2.14.1. RMSD Analysis

**Figure 14:**
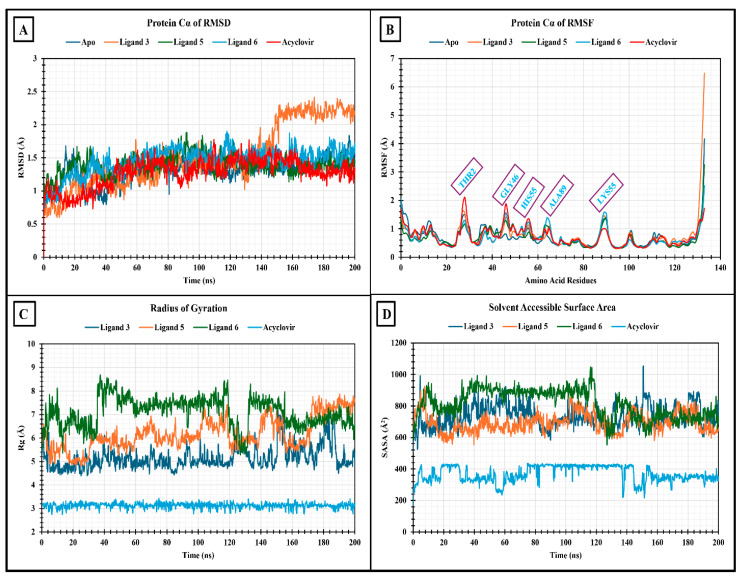
The graph chart plotted above shows the values for RMSD (**A**), RMSF (**B**), Rg (**C**), and SASA (**D**) extracted from the data analysis trajectories by the MD simulation approach for the following ligands: ligand **3** (orange), ligand **5** (green), ligand **6** (sky blue), acyclovir (red), and apoprotein (blue).

A 200 ns MD simulation was performed to examine the conformational change of the target protein in the complex of the desired molecule [26,70]. There were three lead ligands (**3**, **5**, and **6**) and a control (acyclovir) in the complex with an apoprotein (apo) under examination. The lowest and highest RMSD values (Figure 14A) of the apoproteins were 0.656 Å and 1.836 Å, which were observed at frame numbers 4 and 982, respectively. The lowest RMSD values of the three selected ligands (**3**, **5**, and **6**) and the control (acyclovir) were 0.599 Å, 0.907 Å, 0.746 Å, and 0.711 Å in frame numbers 4, 201, 27, and 29, respectively, and the highest were 2.411 Å, 1.882 Å, 1.898 Å, and 1.778 Å in frame numbers 982, 671, 727, and 101, respectively. The average RMSD values of the apoprotein, three ligands (**3**, **5**, and **6**), and the control (acyclovir) were 1.298 Å, 1.509 Å, 1.395 Å, 1.455 Å, and 1.276 Å, respectively. Therefore, ligands **5** and **6** have the nearest RMSD values to the control (acyclovir). These two ligands and apoprotein complexes produced excellent results with no fluctuations. However, the ligand 3–apoprotein complex showed a large conformational change after 150 ns compared with the native structure of the apoprotein. The apoprotein demonstrated good stability, as its average RMSD value was 1.298 Å.

#### 2.14.2. RMSF Analysis

The RMSF can be used to identify and define the local changes in the protein chain that occur when drug-like compounds interact with particular residues [71]. To observe the changes in protein structural flexibility during the attachment of specific molecules to a specific residual position, the RMSF values of ligands **3**, **5**, and **6**, and the control (acyclovir) in complex with the apoprotein were calculated, as shown in Figure 14B. The lowest and highest RMSF values of the apoprotein were 0.323 Å and 4.163 Å, which were observed at residual positions ILE96 and ASN133, respectively. The RMSF values of the three ligands that were selected (**3**, **5**, and **6**) and the control (acyclovir) were as follows: the lowest values were 0.296 Å, 0.324 Å, 0.311 Å, and 0.315 Å, respectively, at residual positions ILE94, VAL95, ILE94, and ILE94; the highest values were 6.476 Å, 3.248 Å, 2.51 Å, and 2.103 Å, respectively, at residual positions THR133, THR133, THR133, and THR28. The average RMSF values of the apoprotein, three ligands (**3**, **5**, and **6**), and the control (acyclovir) were 0.733 Å, 0.773 Å, 0.679 Å, 0.697 Å, and 0.728 Å, respectively. The selected compounds showed a peak region of the protein at the THR28, GLY46, HIS55, LYS65, and ALA89 residual locations, which presented the largest fluctuations during the simulation period.

#### 2.14.3. Radius of Gyration (Rg) Analysis

The stiffness and mobility of proteins are determined by analyzing the Rg of the protein-ligand complex. The calculation of Rg is essential for forecasting the structural activity of macromolecules since it serves as a major predictor of modifications in complex compactness [72]. Consequently, the Rg values of ligands **3**, **5**, and **6**, and acyclovir (control) in association with the target protein were examined throughout the 200 ns simulation duration, as shown in Figure 14C, and 1001 frames were generated, while a 200 ps interval was used for trajectory recording. According to Figure 14C, ligands **3**, **5**, and **6** and acyclovir (control) presented a more steady range of variations, with minimum and maximum values of 4.386 Å in the 124 number frame and 7.383 Å in the 754 number frame (the difference is 2.997 Å), 4.821 Å in the 754 number frame and 7.875 Å in the 754 number frame (the difference is 3.054 Å), 5.22 Å in the 653 number frame and 8.683 Å in the 127 number frame (the difference is 3.463 Å), and 2.741 Å in the 33 number frame and 3.424 Å in the 374 number frame (the difference is 0.683 Å), respectively. For compound ligands **3**, **5**, and **6**, and acyclovir (control), the average Rg values were 5.16 Å, 6.17 Å, 7.10 Å, and 3.12 Å, respectively.

#### 2.14.4. Solvent Accessible Surface Area (SASA)

The SASA was used to determine the function and structure of biomolecules, especially proteins, in their solvent environments. In MD simulations, SASA refers to a measure of the total surface area of a protein exposed by an imaginary solvent sphere center interacting with the molecule’s van der Waals surface [73,74]. It is also used as a parameter to determine the Gibbs energy of protein folding and protein-ligand interactions and identify potential binding sites and solvation effects. An elevated SASA value signifies an expansion in the protein volume, concurrently predicting minimal fluctuations during the simulation period [75,76]. Additionally, a high average SASA value indicates that more amino acid residues are accessible to water molecules and correspond to a high binding interface area. The average SASA values of the protein-ligand complex, as shown in Figure 14D, are 734 Å^2^, 693 Å^2^, 812 Å^2^, and 368 Å^2^ for the ligands; **3**, **5**, and **6** and acyclovir (control) have a system complex ranging from 350 to 850 Å^2^. In addition, our findings show that the lowest SASA values of the three selected ligands (**3**, **5**, and **6**) and the control (acyclovir) were 509 Å^2^, 553 Å^2^, 553 Å^2^, and 166 Å^2^ in frame numbers 0, 199, 635, and 0, respectively, and the highest were 1052 Å^2^, 922 Å^2^, 1045 Å^2^, and 434 Å^2^ in frame numbers 754, 37, 581, and 374, respectively.

### 2.15. Assessment of Protein–Ligand Interactions

**Figure 15:**
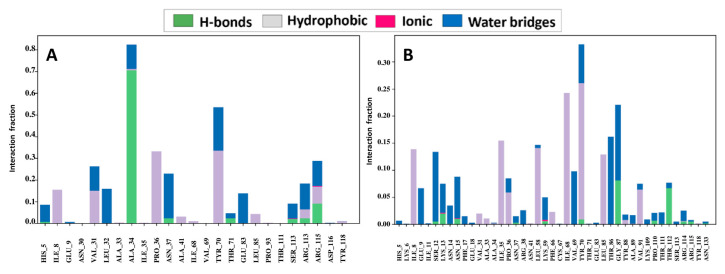

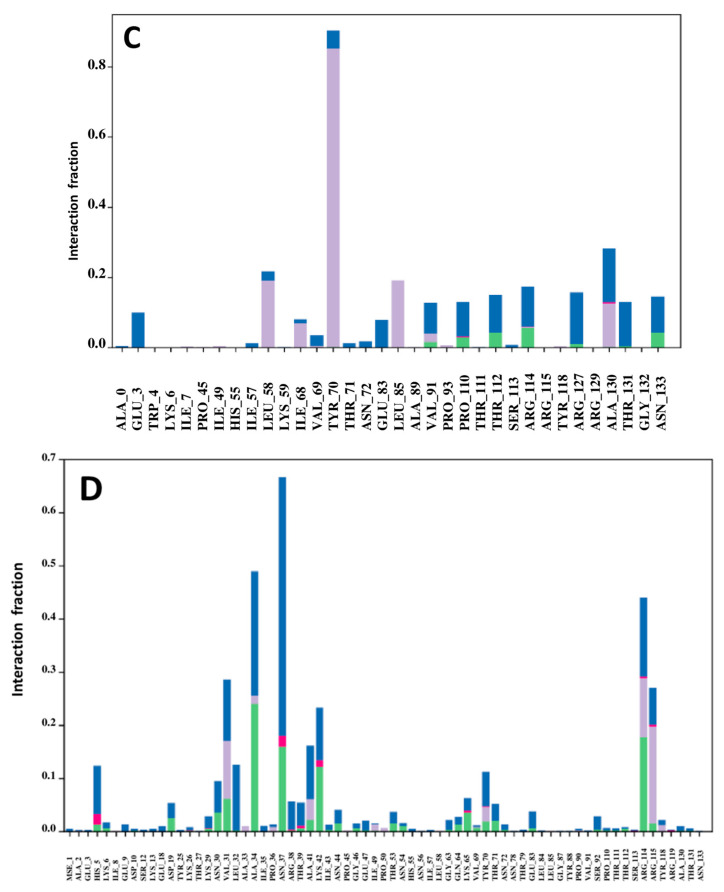
The bar charts illustrate the correlation between the ligand and chain A of the 4QWO protein, as observed during the 200 ns simulation. Therefore, they demonstrated the interaction between the monkeypox virus 4QWO protein and four specific compounds: ligands **3** (**A**), **5** (**B**), and **6** (**C**) and acyclovir (control) (**D**).

A simulation interaction diagram (SID) was used to study a protein’s complex architecture, assigned ligands, and intermolecular interactions during a 200 ns simulation period. The protein–ligand complexes have been evaluated and are presented by employing several bonds, including hydrogen bonds, ionic bonds, noncovalent bonds (hydrophobic bonds), and water bridges [76,77]. Each molecule exhibited a unique interaction during the course of the 200 ns simulation, culminating in the establishment of stable binding with the target protein. At residues ALA34, SER113, ARG114, and ARG115, ligand **3** produced multiple contacts with interaction fractions (IFs) of 0.8, 0.08, 0.19, and 0.28, respectively. As shown in Figure 15, the simulation results in a durable specific connection because the same subtype contacts the ligand on multiple occasions. The residues LYS13 (0.07), ASN15 (0.085), LYS59 (0.045), and ARG114 (0.025) produced multiple contacts with ligand **5** (Figure 15). Ligand **6** had several interactions at residues VAL91 (0.18), PRO110 (0.187), ARG114 (0.0.19), and ALA (0.23). The control molecule, acyclovir, had multiple interactions at residues HIS5 (0.12), THR26 (0.03), LYS29 (0.04), VAL31 (0.28), ALA34 (0.46), ASN37 (0.68), THR39 (0.04), ALA41 (0.14), and LYS42 (0.23).

### 2.16. MD Trajectory Data Analysis of Marburg Virus

#### 2.16.1. RMSD Analysis

**Figure 16:**
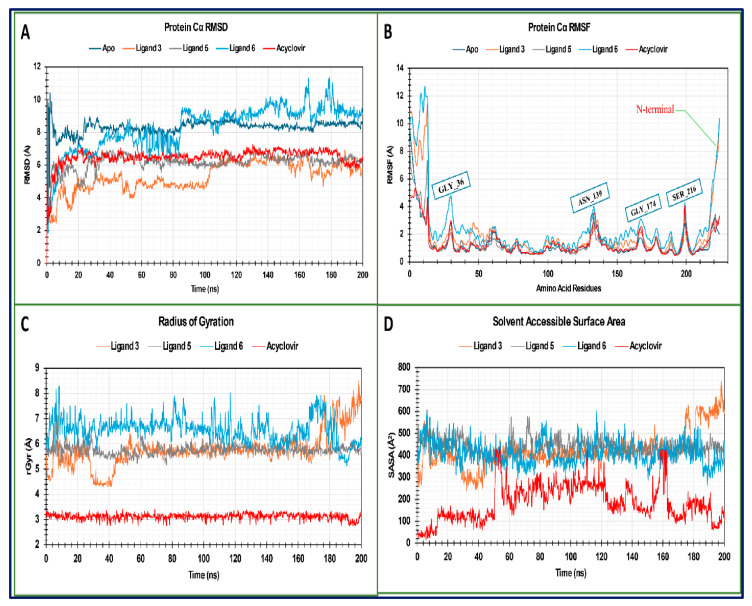
Protein Cα RMSD (**A**), RMSF (**B**), Rg (**C**), and SASA (**D**) were found from a 200 ns MD trajectory through a simulation interaction diagram.

An investigation of the conformational modification of the target protein in the complex of the indicated compound comprising ligands **3**, **5**, and **6** and a positive control, acyclovir, is shown in Figure 16A. The average RMSD values for compound ligands **3**, **5**, and **6** and the control acyclovir when complexed with the apoprotein are 5.29 Å, 6.17 Å, 8.27 Å, and 6.47 Å, respectively. The selected compounds, ligands **3**, **5**, and **6** and the control acyclovir, had the highest RMSD values, as follows: 6.928 Å (frame number 944), 9.793 Å (frame number 2), 11.335 Å (frame number 894), and 7.253 Å (frame number 652). However, the lowest RMSD values, along with their frame numbers, of the three selected lead compound ligands **3**, **5**, and **6** and the control acyclovir were 1.73 Å and 2.04 Å in the first frame, 1.896 Å in the fourth frame, and 2.377 Å in the third frame, respectively. Ligand **3** demonstrated remarkable outcomes, even better than those of the control, when complexed with the apoprotein, resulting in better stability, followed by ligand **5**.

#### 2.16.2. RMSF Analysis

Ligands **3**, **5**, and **6** and acyclovir (control) presented average RMSF values of 2.048 Å, 1.388 Å, 2.501 Å, and 1.367 Å, respectively. The lowest and highest RMSF values of apoprotein were 0.555 Å and 9.764 Å, which were observed at residual positions ALA_93 and ASP_2, respectively. The lowest values were 0.55 Å, 0.492 Å, 0.602 Å, and 0.482 Å for ligands **3**, **5**, and **6**, respectively, and for acyclovir (control), at residual positions GLY_99, VAL_199, PHE_221, and LEU_198; the highest values were 11.238 Å, 8.69 Å, 12.66 Å, and 5.316 Å, respectively, at residual positions PRO_11, ASP_2, ASN_9, and ALA_2. The selected compounds showed a peak region of fluctuations in the protein at the GLY_36, ASN_139, GLY_174, and SER_216 residual locations, as illustrated in Figure 16B. Moreover, the stiffest region of fluctuations was due to the secondary structural elements exhibiting the least flexibility, which ranged from 40 to 130 AA and 140 to 160 AA. Compared with the control, acyclovir, the majority of the chosen compounds were stable, with average values ranging from 2 to 3 Å. This suggests a decrease in the mobility of amino acids within the binding region of the protein in comparison to the other compounds [78].

#### 2.16.3. The Radius of Gyration (rGyr) Analysis

The lowest values of rGyr for ligands **3**, **5**, and **6** and acyclovir (control) were 4.308 Å in frame number 181, 5.187 Å in frame number 382, 5.145 Å in frame number 954, and 2.749 Å in frame number 963, where the highest values were 8.516 Å in frame number 992, 6.456 Å in frame number 308, 8.286 Å in frame number 42, and 3.405 Å in frame number 243, respectively, and the average, the rGyr values were found to be 5.789 Å, 5.759 Å, 6.564 Å, and 3.125 Å, respectively. As a result, ligands **3** and **5** had the closest rGyr values to that of acyclovir (Figure 16C).

#### 2.16.4. Solvent Accessible Surface Area (SASA)

The SASA values for ligands **3**, **5**, and **6** and acyclovir (control) were computed, where the lowest values for these compounds were 240.064 Å^2^ in frame number 161, 346.244 Å^2^ in frame number 277, 263.835 Å^2^ in frame number 945, and 15.713 Å^2^ in frame number 963; the following highest values for these compounds were 736.536 Å^2^ in frame number 990, 577.845 Å^2^ in frame number 359, 604.426 Å^2^ in frame number 32, and 432.712 Å^2^ in frame number 243. The average SASA values for ligands **3**, **5**, and **6** and acyclovir (control) were found to be 423.255 Å^2^, 441.131 Å^2^, 412.083 Å^2^, and 186.006 Å^2^, respectively (Figure 16D). The average SASA value varied from 150 Å^2^ to 450 Å^2^ for the complex system.

### 2.17. Protein–Ligand Contact Analysis

**Figure 17:**
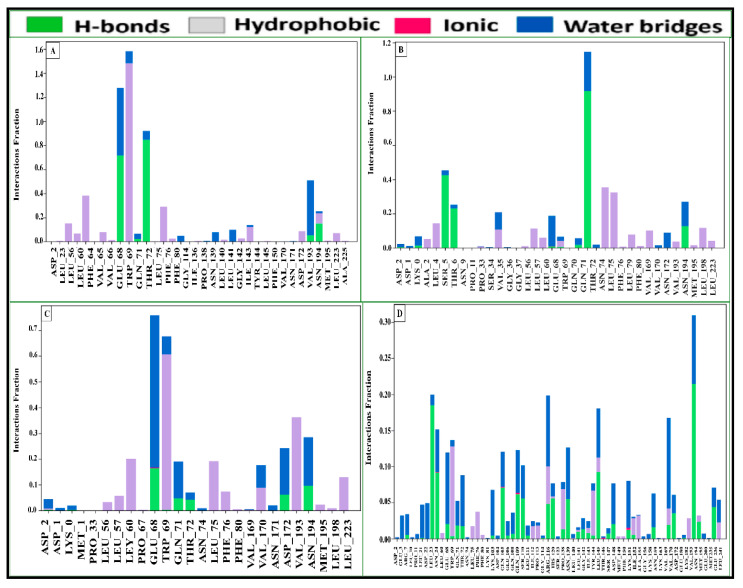
In these bar charts, the 4OR8 protein showed interactions with ligand **3** (**A**), ligand **5** (**B**), ligand **6** (**C**), and acyclovir (**D**) where the P-L interactions were determined during the 200 ns simulation period.

A variety of interactions, such as hydrogen bonds, ionic bonds, noncovalent bonds (hydrophobic bonds), and water bridges, have been used to evaluate and display protein-ligand (**3**, **5**, and **6**, and control acyclovir) contacts. At residue MET195, ligand **3** produced multiple contacts with an interaction fraction (IF) of 0.29. The residues GLU68 (0.19) and TRP69 (0.03) produced multiple contacts with ligand **5**. Ligand **6** had multiple interactions at residues ASP2 (0.04), GLU68 (0.79), and TRP69 (0.65). The control molecule, acyclovir, had multiple interactions at residues ASN24 (0.15), TRP89 (0.14), GLN105 (0.13), GLN109 (0.13), ARG116 (0.20), HIS120 (0.075), PRO138 (0.076), LEU141 (0.02), ILE143 (0.03), TYR144 (0.075), LEU145 (0.17), LYS151 (0.09), ILE152 (0.025), and VAL170 (0.16) (Figure 17).

## 3. Materials and Methods

### 3.1. Materials, Samples, and Equipment

**Figure 18:**
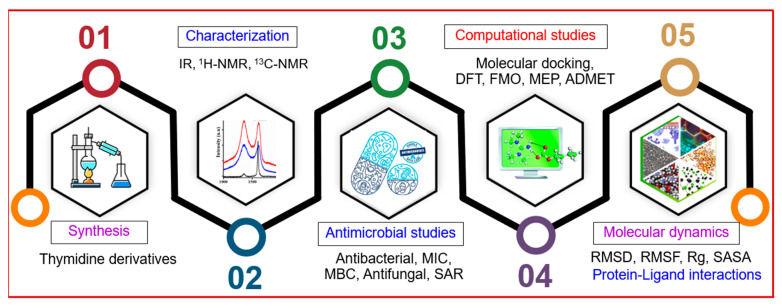
Illustrates the successive steps of conducting the present research.

The standard technique was used to cleanse the analytical solvents. Except where noted, all reagents were obtained from Sigma-Aldrich (Berlin, Germany) and handled in their normal state. A low-pressure Büchi rotary evaporator was used. An FTIR spectrophotometer (Shimadzu, Japan) was used (recoded in KBr) at the University of Chittagong’s Department of Chemistry to acquire infrared measurements. The WMSRC, JU, Bangladesh NMR spectrum recording internal standard was tetramethylsilane. ^1^H and ^13^C spectra were recorded using a Bruker advanced DPX 400 MHz (in CDCl_3_). For Germany’s Kieselgel GF_254_, TLC was used. Chromatograms were obtained by scattering 1% H_2_SO_4_ over plates and heating them to 150–200 °C. A G_60_ silica gel column was used. The bacterial and fungal strains used in this study were obtained from the Department of Microbiology, University of Chittagong. The antimicrobial assay was conducted against five clinically relevant human pathogenic bacteria and two phytopathogenic fungi (Table S1). The current work plan is shown in Figure 18.

### 3.2. Synthesis

#### 3.2.1. The 5′-O-(Palmitoyl)thymidine (**2**)

Cooled thymidine (**1**) (200 mg, 0.82 mmol) in dry pyridine (3 mL) at −5 °C was added before palmitoyl chloride (0.27 mL, 1.1 molar eq.) and the catalyst 4-dimethylaminopyridine (DMAP) were added. First, the mixture was mixed for six hours at this temperature. After that, the mixture was left at RT overnight. The mixture started with methanol and chloroform (1:4) (R*f* = 0.51) and turned into a single product through TLC. The title compound (**2**) (187 mg) was made a white solid by silica gel column chromatography after elution with methanol–chloroform (1:4). The needle-like palmitoyl derivative (**2**) was recrystallized with ethyl acetate-n-hexane.

Yield = 82.18%, m.p. = (72–73) °C, EtOAC-*n*-C_6_H_14_. *Rf* = 0.50, CH_3_OH-CHCl_3_ (1:8 as the mobile phase).

The spectral data are presented in the supplementary data file in the Supplementary Materials.

#### 3.2.2. The General Procedure for the Preparation of Palmitoyl Derivatives (**3–6**)

##### 5′-O-Palmitoyl-3′-O-(pivaloyl)thymidine (**3**)

Compound **2** (92 mg, 0.19 mmol) was chilled to −5 °C and agitated in dry pyridine (3 mL) before pivaloyl chloride (0.051 mL, 2.2 molar eq.) was added. Six hours of room-temperature churning occurred next. TLC (methanol–chloroform 1:8) showed that the starting material changed entirely into a more rapidly moving product (Rf = 0.50). Using methanol–chloroform (1:8) as the eluent for silica gel column chromatography, the **3**-pivaloyl derivative was purified as a crystalline solid (95 mg). From the title product, ethyl acetate-n-hexane was recrystallized into needles (**3**).

Yield = 87.88%, m.p. = (65–66) °C, EtOAC-*n*-C_6_H_14_. *Rf* = 0.53, CH_3_OH-CHCl_3_ (1:9 as the mobile phase).

The spectral data are presented in the supplementary data file in the Supplementary Materials.

Similar reactions and methods of purification were used to prepare lauroyl (**4**) (118 mg, needles), myristoyl (**5**) (61 mg, needles), and 4-t-butylbenzoyl (**6**) (110.9 mg, needles) derivatives.

#### 3.2.3. The 3′-O-Lauroyl-5′-O-(palmitoyl)thymidine (**4**)

Yield = 79.22%, m.p. = (68–69) °C, EtOAC-*n*-C_6_H_14_. *Rf* = 0.52, CH_3_OH-CHCl_3_ (1:8 as the mobile phase).

The spectral data are presented in the supplementary data file in the Supplementary Materials.

#### 3.2.4. The 3′-O-Myristoyl-5′-O-(palmitoyl)thymidine (**5**)

Yield = 88.30%, m.p. = (61–62) °C, EtOAC-*n*-C_6_H_14_. *Rf* = 0.51, CH_3_OH-CHCl_3_ (1:9 as the mobile phase).

The spectral data are presented in the supplementary data file in the Supplementary Materials.

#### 3.2.5. The 3′-O-(4-*t*-Butylbenzoyl)-5′-O-(palmitoyl)thymidine (**6**)

Yield = 79.78%, m.p. = (57–58) °C, EtOAC-*n*-C_6_H_14_. *Rf* = 0.54, CH_3_OH-CHCl_3_ (1:8 as the mobile phase).

The spectral data are presented in the supplementary data file in the Supplementary Materials.

### 3.3. In Vitro Antibacterial Activity Test

The antibacterial activity tests were carried out using a disc diffusion assay. The antibacterial efficacy of the tested agents was assessed using the disk diffusion method in accordance with a previously published protocol with minor modifications [79]. Inoculate bacteria were prepared by suspending bacterial colonies from fresh cultures in 0.9% sterile normal saline, serving as an alternative to the standard 0.5 McFarland turbidity standard. The standardized suspensions were evenly spread onto Mueller–Hinton agar (MHA) plates. Sterile 6 mm Hi-Media filter paper discs containing the test substances were then placed onto the agar surface. The plates were incubated at 35–37 °C for 18 h, and the diameters of the resulting zones of inhibition were measured in millimeters. All the assays were performed in triplicate to ensure the reliability and reproducibility of the results.

### 3.4. Determination of the MIC and MBC via the Broth Microdilution Method

The MIC and MBC were determined via the broth microdilution method, which is recognized as one of the most reliable techniques for quantifying antibacterial activity in vitro [80]. Serial twofold dilutions of the test compound were prepared in columns 2–10 of a sterile 96-well microtiter plate to achieve final concentrations ranging from 0.125 to 128 µg/mL. The first well contained 100 µL of Mueller–Hinton broth (MHB) as a diluent control. Each well received 100 µL of the corresponding 2× diluted compound. A standardized inoculum of 5 µL, adjusted to a 0.5 McFarland turbidity standard, was added to all the wells except those designated sterile controls. Well 11 served as a growth control (inoculated but without the test compound), whereas well 12 served as a sterile control (containing only media). To prevent evaporation and desiccation, the plates were sealed with cling film and incubated at 37 °C for 24 h. Subsequently, 10 µL of 0.5% (*w*/*v*) 2,3,5-triphenyl tetrazolium chloride (TTC) was added to each well and incubated for an additional 30 min at 35–37 °C. A red color change indicated bacterial growth, allowing visual determination of the MIC and MBC values.

### 3.5. Determination of Mycelial Growth

The antifungal activity of the test compounds was evaluated using the poisoned food technique on potato dextrose agar (PDA) as previously described [81] with minor modifications; the compounds were dissolved in 1% (*w*/*v*) dimethyl sulfoxide (DMSO), and 0.1 mL of each solution, equivalent to 1 mg of the test compound, was aseptically transferred onto sterile Petri dishes. Subsequently, 20 mL of molten PDA was poured into each dish and allowed to solidify. A 5 mm diameter mycelial disc from an actively growing fungal culture was aseptically placed at the center of each plate, ensuring direct contact between the mycelium and the test medium by inverting the inoculum disc. All the assays were conducted in triplicate and incubated at 27 ± 2 °C. Control plates containing PDA without the test compounds (DMSO only) were maintained under identical conditions. Fungal radial mycelial growth was measured after five days of incubation, and the average diameter of fungal colonies (in mm) was calculated from three independent replicates.

### 3.6. Structure–Activity Relationship

SAR analysis was employed to identify potential antibacterial pharmacological targets on the basis of the molecular structures of the test compounds. This widely adopted approach in rational drug design facilitates the optimization and development of novel agents by correlating structural features with biological activity. In the present study, SAR analysis was guided by the previously proposed concept of cell membrane permeability [82], which serves as a key criterion for evaluating and predicting antibacterial efficacy.

### 3.7. Computational Details

Compounds (**1**–**6**) were optimized via Gaussian 09 [83] software at the DFT/B3LYP/6-311+G(d,p) level of theory. The 3D graphs are taken from the GaussView 6 [84] and Vesta 6 [85] interfaces. The nucleophilic and electrotrophic sites in each compound were studied by MEP analysis. In addition, orbital occupancies and acceptor groups that are useful for the attack of selected viruses were investigated. The FMO iso-surfaces were calculated via TD-DFT/B3LYP6-311+G(d,p). The reactivity parameters are discussed in detail for each compound. Finally, the NBO charge provides information on the major electronic population occupancy on the surface of each material.

### 3.8. Protein Selection and Molecular Docking

The AutoDock 4.2 program [86] enabled the molecular docking simulations (www.rcsb.org) (accessed 10 November 2024). The crystal structures of the A42R profilin-like protein from the monkeypox virus Zaire-96-I-16 (PDB: 4QWO) [87] and Marburg virus VP24 (PDB: 4OR8) [88] were downloaded from the RSCB protein database (13 November 2024). The water molecules were first removed, and then the system was adjusted to incorporate Gasteiger charges and missing hydrogens during receptor input file construction. Every ligand and protein file PDBQT was created via AutoDock Tools. Auto Grid precalculates the grid maps, therefore saving a great deal of time for docking studies. All the compound geometries were optimized via ACD (3D viewer) software (http://www.filefacts.com/acd3d-viewer-freeware-info) (version 4.5). Discovery Studio 2017R2 (https://www.3ds.com/products/biovia/discovery-studio) (accessed 12 December 2024) allows interactions to be seen and examined.

### 3.9. ADMET Properties Involving Pharmacokinetic Prediction

The ADMET properties involving pharmacokinetic studies of the newly synthesized molecules **2**–**6** were estimated through the pkCSM online server (https://biosig.lab.uq.edu.au/pkcsm/prediction).

### 3.10. Molecular Dynamics Simulation

MD simulations were used to examine the structural stability of protein-ligand complexes under particular physiological circumstances [89]. Currently, only the classical MD simulation approach can be employed to simulate biomolecular systems consisting of hundreds of atoms and covering periods ranging from picoseconds to nanoseconds in duration [90]. Initially, our objective was to identify the top three compounds with the best docking scores, which we did, and then, the data were subjected to preprocessing via protein preparation in Wizard’s format for the following compounds: ligand **3**, ligand **5**, ligand **6**, and acyclovir (control), and PDB ID: 4WQO (monkeypox) and 4OR8 (Marburg) proteins. Complexes are generated from AutoDock output files. The Desmond package from the Schrödinger suite was then used to perform a 200 ns molecular dynamics simulation. To maintain consistent system volumes, each complex SPC water model has an orthorhombic periodic boundary box shape with a distance of 10 × 10 × 10 Å^3^. Na^+^ and Cl^−^ ions were distributed randomly throughout the solvated system to maintain a salt concentration of 0.15 M, and the system was relaxed via the OPLS-3e force field. The ensemble was characterized by NPT conditions (fixed particle count, temperature, and pressure), where the particles were kept at a static temperature of 300 K and a pressure of 1.0 atmospheric (1.01325 bar) intervals of 200 ns with an energy of 1.2 recorded. During this time, the solvent and ions were equally distributed around the protein-ligand complex [91]. To evaluate each complex’s dynamic and steady properties, we allowed the system to relax and then recorded the data trajectories to visualize the results by RMSD, RMSF, SASA, Rg, and protein-ligand contact, which were subsequently calculated [92].

## 4. Conclusions and Future Perspectives

This study investigated the antibacterial, antifungal, SAR, global reactivity, molecular docking, dynamic, protein-ligand interaction, and ADMET properties of thymidine derivatives (**2–6**) via in vitro and in silico approaches. Three-dimensional MEP analysis indicated that compounds **5** and **6** exhibit strong binding potential against specific target proteins. Compared with the parent molecule, most of the thymidine derivatives displayed a reduced HOMO-LUMO gap, suggesting enhanced chemical reactivity. The introduction of aliphatic and aromatic groups into the deoxyribose scaffold significantly improved the biological activity. Notably, the lauroyl (C12) and myristoyl (C14) derivatives (**4** and **5**) demonstrated superior pharmacokinetic profiles and antimicrobial efficacy against both bacterial and fungal strains. Molecular docking studies revealed promising antiviral potential for these thymidine derivatives, with 200 ns MD simulations confirming the stability of derivative **5** against monkeypox and Marburg virus proteins. Protein-ligand interactions remained stable throughout the simulations, supporting their biological relevance. ADMET predictions indicated favorable pharmacokinetic properties, with all the compounds meeting the drug-likeness criteria. High Caco-2 permeability (>30%) suggested efficient intestinal absorption. Given the combined synthetic, antimicrobial, and computational evidence, these findings highlight the therapeutic potential of thymidine derivatives. However, further in vitro and in vivo validation is needed before clinical consideration.

## Figures and Tables

**Table 1:** Compounds **1**–**6** demonstrated a zone of inhibition against microorganisms.

	Diameter of Inhibition Zone (mm)				
Entry	*B. subtilis (+ve)*	*B. cereus (+ve)*	*E. coli* *(−ve)*	*S. typhi* *(−ve)*	*P. aeruginosa* *(−ve)*
**1**	NI	NI	NI	NI	NI
**2**	NI	NI	7.84 ± 0.1	NI	NI
**3**	NI	NI	7.75 ± 0.2	NI	NI
**4**	* 11.00 ± 0.1	* 17.00 ± 0.4	* 20.75 ± 0.2	9.75 ± 0.2	* 17.75 ± 0.2
**5**	* 10.00 ± 0.2	* 15.00 ± 0.2	9.26 ± 0.2	8.13 ± 0.3	* 14.00 ± 0.1
**6**	NI	NI	NI	NI	NI
Azithromycin	18.25 ± 0.3 **	17.75 ± 0.3 **	** 17.25 ± 0.1	** 18.00 ± 0.2	** 18.5 ± 0.3

Data represent mean ± SD from three independent experiments. *p* < 0.05 indicates significant inhibition; (*) denotes marked inhibition; (**) represents azithromycin control; NI = no inhibition.

**Table 2:** Percent inhibition of antifungal activity by the synthesized compounds.

Entry	% Fungal Mycelial Growth Inhibition in mm (20 μg/μL)	
	*Aspergillus niger*	*Aspergillus* *flavus*
**1**	NI	NI
**2**	* 73.72 ± 1.2	* 77.45 ± 1.0
**3**	* 77.54 ± 1.1	NI
**4**	* 64.40 ± 1.3	* 74.59 ± 1.2
**5**	55.61 ± 1.1	* 61.52 ± 1.0
**6**	NI	NI
Nystatin	** 65.4 ± 1.0	** 64.1 ± 1.0

Data represent mean ± SD from three independent experiments. *p* < 0.05 indicates significant inhibition; (*) denotes marked inhibition; (**) represents nystatin control; NI = no inhibition.

**Table 3:** Global reactivity parameters of all studied compounds (**1**–**6**) calculated at the TD-DFT/B3LYP/6-311+G(d, p) level of theory.

	HOMO	LUMO	χ (eV)	η (eV)	S	μ (eV)	ω (eV)
**1**	−6.83	−1.52	4.17	2.65	0.37	−4.17	3.28
**2**	−6.64	−1.33	3.98	2.65	0.37	−3.98	2.98
**3**	−6.60	−1.27	3.93	2.66	0.37	−3.93	2.90
**4**	−7.25	−1.84	4.54	2.70	0.37	−4.54	3.81
**5**	−6.67	−1.34	4.00	2.66	0.37	−4.00	3.00
**6**	−6.73	−2.05	4.39	2.34	0.42	−4.39	4.11

**Table 4:** Docking results of the docked compounds in the binding cavity of the A42R profilin-like protein from the monkeypox virus.

Ligand	Binding Affinity (kcal/mol)	Number of H-Bonds Formed
**1**	−4.5	0
**2**	−4.6	1
**3**	−5.1	1
**4**	−5.0	1
**5**	−6.2	2
**6**	−5.7	1
Reference (acyclovir)	−4.2	2

**Table 5:** Docking results of the docked compounds in the binding cavity of Marburg virus.

Ligand	Binding Affinity (kcal/mol)	Number of H-Bonds Formed
**1**	−4.9	1
**2**	−5.3	1
**3**	−5.5	1
**4**	−5.3	1
**5**	−5.6	2
**6**	−7.0	3
Reference (acyclovir)	−4.3	1

**Table 6:** Pharmacokinetic properties of target compounds **2–6**.

	2	3	4	5	6
Water solubility (log mol/L)	−5.061	−2.892	−5.4	−3.597	−2.892
Caco-2 permeability (log Papp in 10^−6^ cm/s)	0.53	3.008	0.703	0.185	3.008
Intestinal absorption (% Absorbed)	68.559	69.881	77.117	71.954	69.881
Skin permeability (log Kp)	−2.767	−2.735	−2.714	−2.735	−2.735
VDss (log L/kg)	−0.732	0.011	−0.738	−1.203	0.011
Fraction unbound (Fu)	0.137	0.381	0	0.089	0.381
BBB permeability (log BB)	−1.283	−0.245	−1.254	−1.784	−0.245
CNS permeability (log PS)	−3.409	−1.488	−2.85	−3.782	−1.488
CYP2D6 substrate	No	No	No	No	No
CYP3A4 substrate	Yes	No	Yes	Yes	No
CYP1A2 inhibitor	No	Yes	No	No	Yes
CYP2C19 inhibitor	No	No	No	No	No
CYP2C9 inhibitor	No	No	No	No	No
CYP2D6 inhibitor	No	No	No	No	No
CYP3A4 inhibitor	No	No	No	No	No
Renal OCT2 substrate	No	No	No	No	No
AMES toxicity	No	Yes	No	No	Yes
Max. tolerated dose (log mg/kg/day)	−0.2	0.438	0.069	0.168	0.438
Hepatotoxicity	Yes	No	Yes	Yes	No
hERG I inhibitor	No	No	No	No	No
hERG II inhibitor	No	No	No	No	No

## Data Availability

Data is contained within the article.

## Supplementary Materials

The following supporting information can be downloaded at https://www.mdpi.com/article/10.3390/ph18060806/s1, Figures S1–S11: FTIR, 1H-NMR and 13C-NMR spectra of the compounds (2–6); Figures S12 and S13: Antibacterial and antifungal inhibition of the compounds against tested pathogens; Table S1: Name of the pathogenic organisms; Tables S2 and S3: MIC and MBC values of the compounds against tested organisms.

## Abbreviations

The following abbreviations are used in this manuscript:

ADMET	absorption, distribution, metabolism, excretion, and toxicity
DFT	density functional theory
FMO	frontier molecular orbital
HOMO	highest occupied molecular orbital
LUMO	lowest unoccupied molecular orbital
MBC	minimum bactericidal concentration
MD	molecular dynamics
MEP	molecular electrostatic potential
MIC	minimum inhibitory concentration
NBO	natural bond charge
PASS	prediction of substance activity spectra
SAR	structure–activity relationship
