# Supplementary material for: Unveiling Palmitoyl Thymidine Derivatives as Antimicrobial/Antiviral Inhibitors: Synthesis, Molecular Docking, Dynamic Simulations, ADMET, and Assessment of Protein–Ligand Interactions
Source: Pharmaceuticals (Basel). 2025 May 27;18(6):806. doi: 10.3390/ph18060806 (PMC12196243; doi:10.3390/ph18060806)
Supplement: Supplementary file 1 [file pharmaceuticals-18-00806-s001.zip › pharmaceuticals-3649173-supplementary.pdf]

Supplementary Material

Unveiling Palmitoyl Thymidine Derivatives as Antimicrobial/Antiviral Inhibitors: Synthesis, Molecular Docking, Dynamic Simulations, ADMET and Assessment of Protein-Ligand Interactions

#
#
##

Table of Contents

1. FTIR, ^{1}H and ^{13}C NMR spectral data of compounds (2-6)........................2
2. FTIR spectra of compounds (2-6) 5
3. ^{1}H and ^{13}C NMR spectra of compounds (2-6)..6
4. Biological data..16
5. Tables 17

1. FTIR, ^{1}H and ^{13}C NMR Spectral data of compounds (2-6)

*2.2.1. 5′-O-(Palmitoyl)thymidine (**2**)*

FTIR: ν_{max} 1702 (-CO), 3406~3501 (br) (-OH), 3280 (-NH) cm^{-1}. ^{1}H-NMR (400 MHz, $CDCl_3$): δ_H 9.02 (1H, s, -NH), 7.28 (1H, d, J = 1.8 Hz, H-6), 5.80 (1H, t, J = 6.4 Hz, H-1′), 5.0 (1H, dd, J = 12.2 and 4.6 Hz, H-5′a), 4.92 (1H, dd, J = 12.1 and 3.7 Hz, H-5′b), 4.30 (1H, m, H-3′), 4.21 (1H, ddd, J = 3.8, 4.8 and 3.8 Hz, H-4′), 3.15 (1H, br s, 3′-OH), 3.88 (1H, ddd, J = 13.6, 6.5 and 4.5 Hz, H-2′a), 3.75 (1H, ddd, J = 13.6, 6.7 and 6.9 Hz, H-2′b), 2.32 {2H, m, $CH_3(CH_2)_{13}\mathit{CH_2}CO$-}, 1.95 (3H, d, J = 1.6 Hz, 5-CH_3), 1.68 {2H, m, $CH_3(\mathit{CH_2})_{12}\mathit{CH_2}CH_2CO$-}, 1.29 {26H, m, $CH_3(\mathit{CH_2})_{13}CH_2CO$-}, 0.90 {3H, m, $\mathit{CH_3}(CH_2)_{14}CO$-}. ^{13}C-NMR (100 MHz, $CDCl_3$): δ_C 172.21 {$CH_3(CH_2)_{14}\mathit{C}O$-}, 169.30 (C-1), 155.10 (C-4), 139.45 (C-3), 114.75(C-2), 89.80 (C-1′), 87.20 (C-4′), 73.25 (C-3′), 64.25 (C-5′), 40.50 (C-2′), 16.10 (C-5), 34.43, 34.38, 31.95, 31.91(×2), 29.52, 29.31, 29.11, 25.11 (×2), 24.77, 22.61, 21.54, 20.01 {$CH_3(\mathit{C}H_2)_{14}CO$-}, 14.11 {$\mathit{C}H_3(CH_2)_{14}CO$-}. LC-MS $[M+1]^+$ 481.60. Calcd. for $C_{25}H_{44}O_5N_2CO$: C=65.01, H=9.17; found: C=65.02, H=9.18%.

*5′-O-Palmitoyl-3′-O-(pivaloyl)thymidine (**3**)*

FTIR: ν_{max} 1720 (-CO), 3297 (-NH) cm^{-1}. ^{1}H-NMR (400 MHz, $CDCl_3$): δ_H 8.99 (1H, s, -NH), 7.28 (1H, d, J = 1.8 Hz, H-6), 5.93 (1H, t, J = 6.6 Hz, H-1′), 5.88 (1H, m, H-3′), 5.61 (1H, dd, J = 12.0 and 4.5 Hz, H-5′a), 5.47 (1H, dd, J = 12.2 and 3.6 Hz, H-5′b), 5.0 (1H, m, H-4′), 3.74 (1H, ddd, J = 13.6, 6.6 and 4.6 Hz, H-2′a), 2.37 {2H, m, $CH_3(CH_2)_{13}\mathit{CH_2}CO$-}, 2.33 (1H, ddd, J = 12.5, 6.1 and 6.2 Hz, H-2′b), 1.95 (3H, d, J = 1.3 Hz, 5-CH_3), 1.65 {2H, m, $CH_3(\mathit{CH_2})_{12}\mathit{CH_2}CH_2CO$-}, 1.32 {9H, s, $(\mathit{CH_3})_3CCO$-}, 1.29 {26H, m, $CH_3(\mathit{CH_2})_{13}CH_2CO$-}, 0.89 {3H, m, $\mathit{CH_3}(CH_2)_{14}CO$-}. ^{13}C-NMR (100 MHz, $CDCl_3$): δ_C 176.90 {$(CH_3)_3C\mathit{C}O$-}, 172.24 {$CH_3(CH_2)_{14}\mathit{C}O$-}, 169.32 (C-1), 155.11 (C-4), 139.42 (C-3), 114.76 (C-2), 89.88 (C-1′), 87.23 (C-4′), 73.29 (C-3′), 64.26 (C-5′), 40.52 (C-2′), 16.13 (C-5), 38.88 {$(CH_3)_3\mathit{C}CO$-}, 34.44, 34.35, 31.96, 31.92 (×2), 29.55, 29.34, 29.12, 25.15 (×2), 24.70, 22.62, 21.55, 20.02 {$CH_3(\mathit{C}H_2)_{14}CO$-}, 27.11, 26.94, 26.86 {$(\mathit{C}H_3)_3CCO$-}, 14.10 {$\mathit{C}H_3(CH_2)_{14}CO$-}. LC-MS $[M+1]^+$ 564.78. Calcd. for $C_{30}H_{52}O_6N_2CO$: C=65.98, H=9.22; found: C=65.97, H=9.23%.

*2.2.3. 3′-O-Lauroyl-5′-O-(palmitoyl)thymidine (**4**)*

FTIR: ν_{max} 1715 (-CO), 3298 (-NH) cm^{-1}. ^{1}H-NMR (400 MHz, $CDCl_3$): δ_H 9.0 (1H, s, -NH), 7.27(1H, d, J = 1.8 Hz, H-6), 6.33 (1H, t, J = 6.4 Hz, H-1′), 5.59 (1H, m, H-3′), 5.36 (1H, dd, J = 12.1 and 4.5 Hz, H-5′a), 5.28 (1H, dd, J = 12.0 and 3.6 Hz, H-5′b), 5.09 (1H, ddd, J = 3.8, 4.8 and 3.8 Hz, H-4′), 2.69 (1H, ddd, J = 13.4, 6.4 and 4.4 Hz, H-2′a), 2.43 {2H, m, $CH_3(CH_2)_9C\mathit{H}_2CO$-}, 2.36 {2H, m, $CH_3(CH_2)_{13}C\mathit{H}_2CO$-}, 2.13 (1H, ddd, J = 13.5, 6.6 and 6.8 Hz, H-2′b), 1.94 (3H, d, J = 1.6 Hz, 5-CH_3), 1.68 {2H, m, $CH_3(CH_2)_8C\mathit{H}_2CH_2CO$-}, 1.62 {2H, m, $CH_3(C\mathit{H}_2)_{12}C\mathit{H}_2CH_2CO$-}, 1.29 {16H, m, $CH_3(C\mathit{H}_2)_8CH_2CH_2CO$-}, 1.27 {26H, m, $CH_3(C\mathit{H}_2)_{13}CH_2CO$-}, 0.91 {3H, m, $C\mathit{H}_3(CH_2)_{10}CO$-}, 0.90 {3H, m, $C\mathit{H}_3(CH_2)_{14}CO$-}. ^{13}C-NMR (100 MHz, $CDCl_3$): δ_C 172.46 {$CH_3(CH_2)_{10}\mathit{C}O$-}, 172.23 {$CH_3(CH_2)_{14}\mathit{C}O$-}, 169.32 (C-1), 155.16 (C-4), 139.44 (C-3), 114.76 (C-2), 89.83 (C-1′), 87.22 (C-4′), 73.29 (C-3′), 64.23 (C-5′), 40.54 (C-2′), 16.12 (C-5), 34.43, 34.36, 31.97, 31.92 (×2), 29.53, 29.34, 29.17, 25.10 (×2), 24.79, 22.60, 21.52, 20.01 {$CH_3(\mathit{C}H_2)_{14}CO$-}, 34.37, 31.90, 29.52 (×2), 29.10, 25.08 (×2), 22.05, 21.21, 20.11 {$CH_3(\mathit{C}H_2)_{10}CO$-}, 14.10 {$\mathit{C}H_3(CH_2)_{14}CO$-}, 13.49 {$\mathit{C}H_3(CH_2)_{10}CO$-}. LC-MS $[M+1]^+$ 663.95. Calcd. For $C_{37}H_{66}O_6N_2CO$: C=68.78, H=9.95; found: C=68.79, H=9.97%.

*2.2.4. 3′-O-Myristoyl-5′-O-(palmitoyl)thymidine (**5**)*

FTIR: ν_{max} 1725 (-CO), 3299 (-NH) cm^{-1}. ^{1}H-NMR (400 MHz, $CDCl_3$)**:** δ_H 9.0 (1H, s, -NH), 7.26 (1H, d, J = 1.3 Hz, H-6), 6.22 (1H, t, J = 6.5 Hz, H-1′), 5.55 (1H, m, H-3′), 5.45 (1H, dd, J = 12.0 and 4.5 Hz, H-5′a), 5.20 (1H, dd, J = 12.0 and 3.5 Hz, H-5′b), 4.0 (1H, ddd, J = 3.5, 4.5 and 3.9 Hz, H-4′), 2.39 (1H, ddd, J = 13.5, 6.5 and 4.0 Hz, H-2′a), 2.36 {2H, m, $CH_3(CH_2)_{11}C\mathit{H}_2CO$-}, 2.33 {2H, m, $CH_3(CH_2)_{13}C\mathit{H}_2CO$-}, 2.27 (1H, ddd, J = 13.5, 6.5 and 6.7 Hz, H-2′b), 1.95 (3H, d, J = 1.3 Hz, 5-CH_3), 1.66 {2H, m, $CH_3(CH_2)_{10}C\mathit{H}_2CH_2CO$-}, 1.64 {2H, m, $CH_3(C\mathit{H}_2)_{12}C\mathit{H}_2CH_2CO$-}, 1.32 {20H, m, $CH_3(C\mathit{H}_2)_{10}CH_2CH_2CO$-}, 1.29 {26H, m, $CH_3(C\mathit{H}_2)_{13}CH_2CO$-}, 0.92 {3H, t, J = 6.6 Hz, $C\mathit{H}_3(CH_2)_{12}CO$-}, 0.91 {3H, m, $C\mathit{H}_3(CH_2)_{14}CO$-}. ^{13}C-NMR (100 MHz, $CDCl_3$): δ_C 172.54 {$CH_3(CH_2)_{12}\mathit{C}O$-}, 172.20 {$CH_3(CH_2)_{14}\mathit{C}O$-}, 169.28 (C-1), 155.14 (C-4), 139.44 (C-3), 114.77 (C-2), 89.88 (C-1′), 87.23 (C-4′), 73.27 (C-3′), 64.26 (C-5′), 40.51 (C-2′), 16.16 (C-5), 34.44, 34.37, 31.96, 31.90 (×2), 29.51, 29.35, 29.10, 25.13 (×2), 24.76, 22.60, 21.55, 20.02 {$CH_3(\mathit{C}H_2)_{14}CO$-}, 34.38, 34.12 (×2), 31.92, 29.59 (×2), 29.15, 24.96, 21.72 (×2), 20.09 (×2) {$CH_3(\mathit{C}H_2)_{12}CO$-},14.12 {$\mathit{C}H_3(CH_2)_{14}CO$-}, 14.01 {$\mathit{C}H_3(CH_2)_{12}CO$-}. LC-MS $[M+1]^+$ 692.01. Calcd. for $C_{39}H_{70}O_6N_2CO$: C=69.64, H=10.13 found: C=69.63, H=10.14%.

*2.2.5. 3′-O-(4-t-Butylbenzoyl)-5′-O-(palmitoyl)thymidine (**6**)*

FTIR: ν_{max} 1722 (-CO), 3298 (-NH) cm^{-1}. ^1H-NMR (400 MHz, $CDCl_3$): δ_H 9.0 (1H, s, -NH), 8.03 (2H, m, Ar-H), 7.51 (2H, m, Ar-H), 7.29 (1H, d, J = 1.8 Hz, H-6), 6.38 (1H, t, J = 6.4 Hz, H-1′), 5.54 (1H, m, H-3′), 5.16 (1H, dd, J = 12.1 and 4.5 Hz, H-5′a), 3.86 (1H, dd, J = 12.0 and 3.6 Hz, H-5′b), 3.57 (1H, ddd, J = 3.8, 4.8 and 3.8 Hz, H-4′), 3.02 (1H, ddd, J = 13.4, 6.4 and 4.4 Hz, H-2′a), 2.47 {2H, m, $CH_3(CH_2)_{13}CH_2CO$-}, 2.37 (1H, ddd, J = 13.5, 6.6 and 6.8 Hz, H-2′b), 1.95 (3H, d, J = 1.6 Hz, 5-CH_3), 1.66 {2H, m, $CH_3(CH_2)_{12}CH_2CH_2CO$-}, 1.31, {9H, s, $(CH_3)_3C$-}, 1.27 {26H, m, $CH_3(CH_2)_{13}CH_2CO$-}, 0.91 {3H, m, $CH_3(CH_2)_{14}CO$-}. ^{13}C-NMR (100 MHz, $CDCl_3$): δ_C 174.40 {$(CH_3)_3CC_6H_4CO$-}, 172.27 {$CH_3(CH_2)_{14}CO$-}, 169.35 (C-1), 155.17 (C-4), 139.43 (C-3), 132.44, 132.40, 130.94, 129.91, 126.52, 125.50 {$(CH_3)_3CC_6H_4CO$-}, 114.74 (C-2), 89.87 (C-1′), 87.25 (C-4′), 73.27 (C-3′), 64.20 (C-5′), 40.53 (C-2′), 16.14 (C-5), 34.45, 34.34, 31.97, 31.94 (×2), 29.53, 29.34, 29.15, 25.16 (×2), 24.72, 22.68, 21.55, 20.02 {$CH_3(CH_2)_{14}CO$-}, 35.67 {$(CH_3)_3CC_6H_4CO$-}, 14.13 {$CH_3(CH_2)_{14}CO$-}, 13.67, 13.65, 13.42 {$(CH_3)_3CC_6H_4CO$-}. LC-MS $[M+1]^+$ 641.86. Calcd. for $C_{36}H_{56}O_6N_2CO$: C=69.28, H=8.73; found: C=69.30, H=8.74%.

2. FTIR spectra of compounds (2-6)

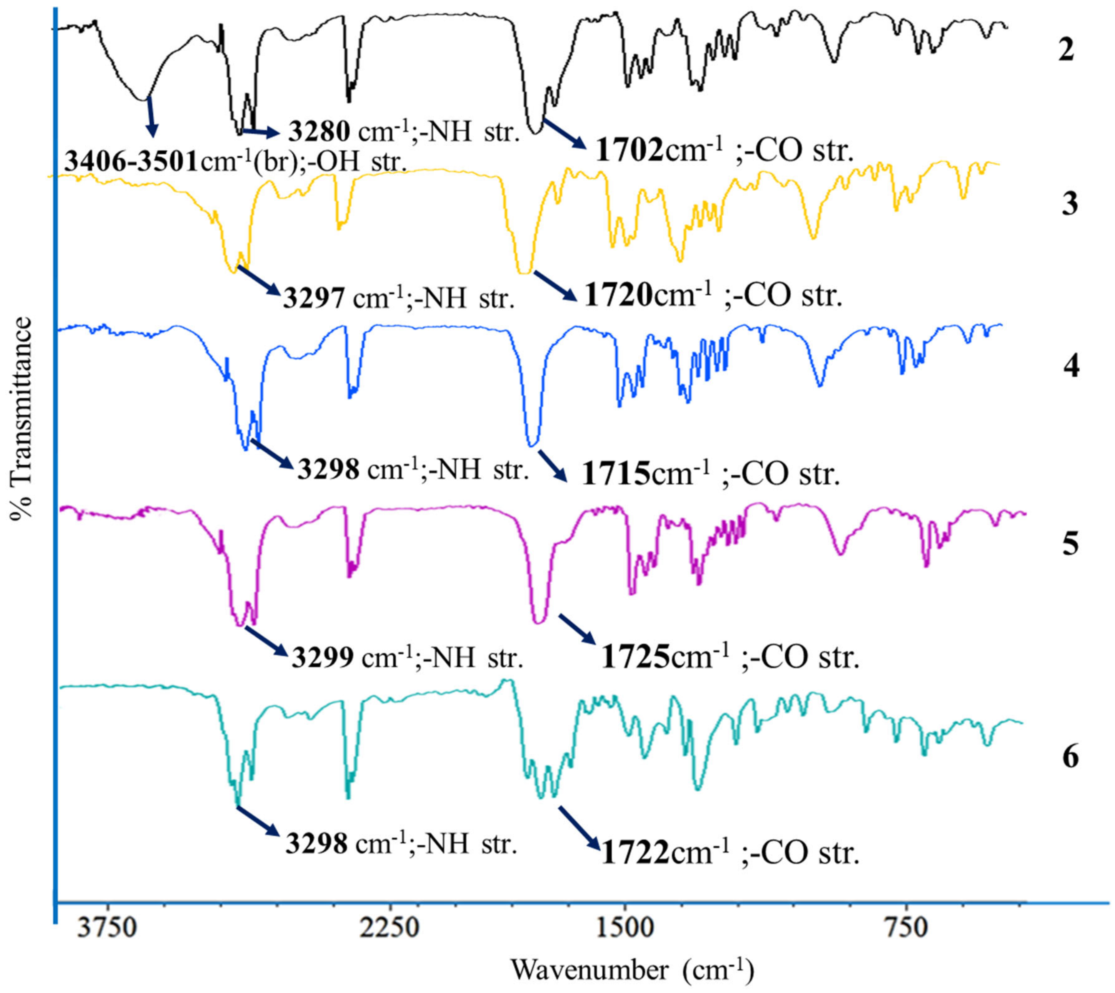

Figure S1. FTIR spectra of the compound (**2-6**).

3. ^{1}H and ^{13}C NMR spectra of compounds (2-6)

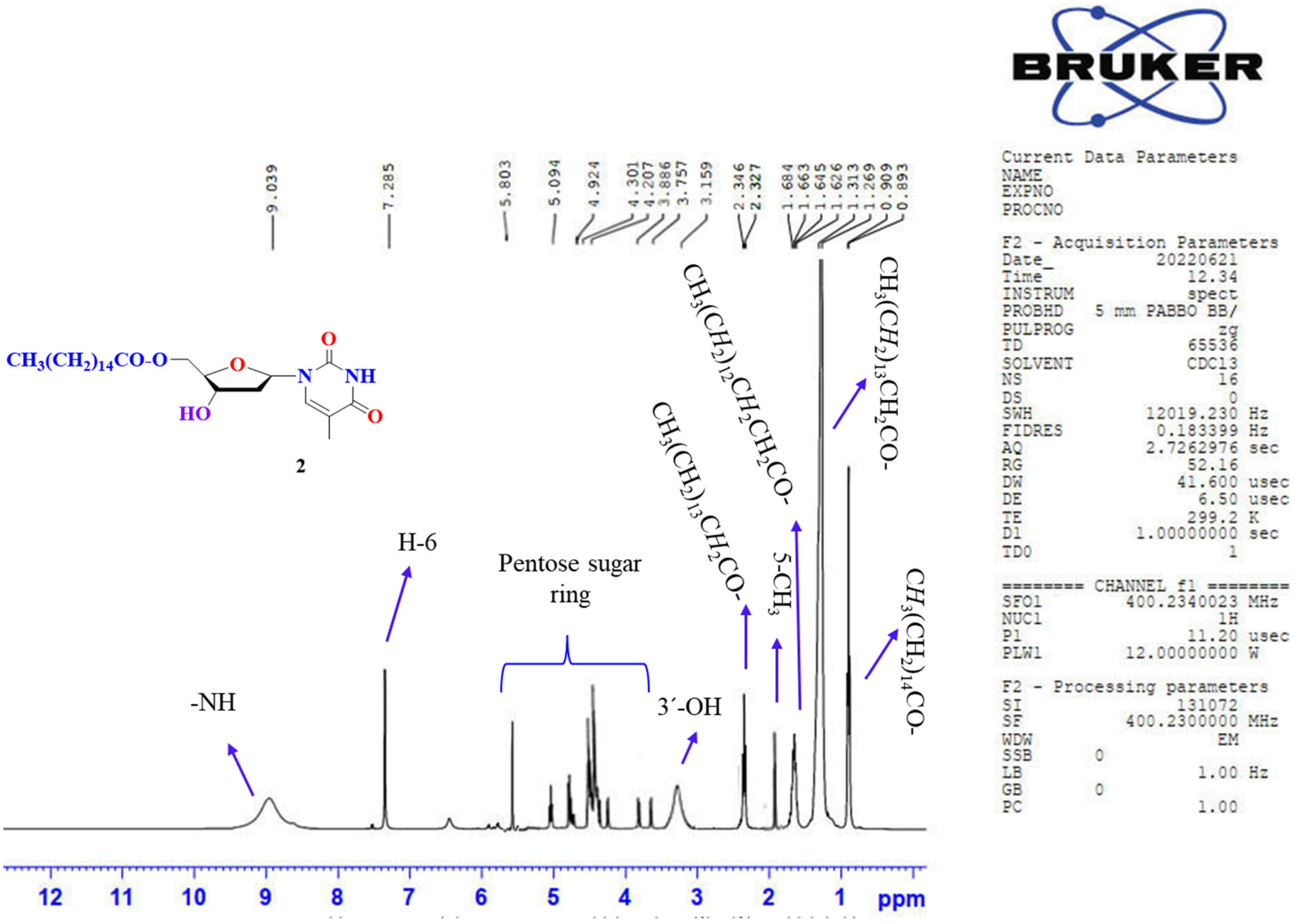

Figure S2. ^{1}H-NMR spectra of the compound (**2**).

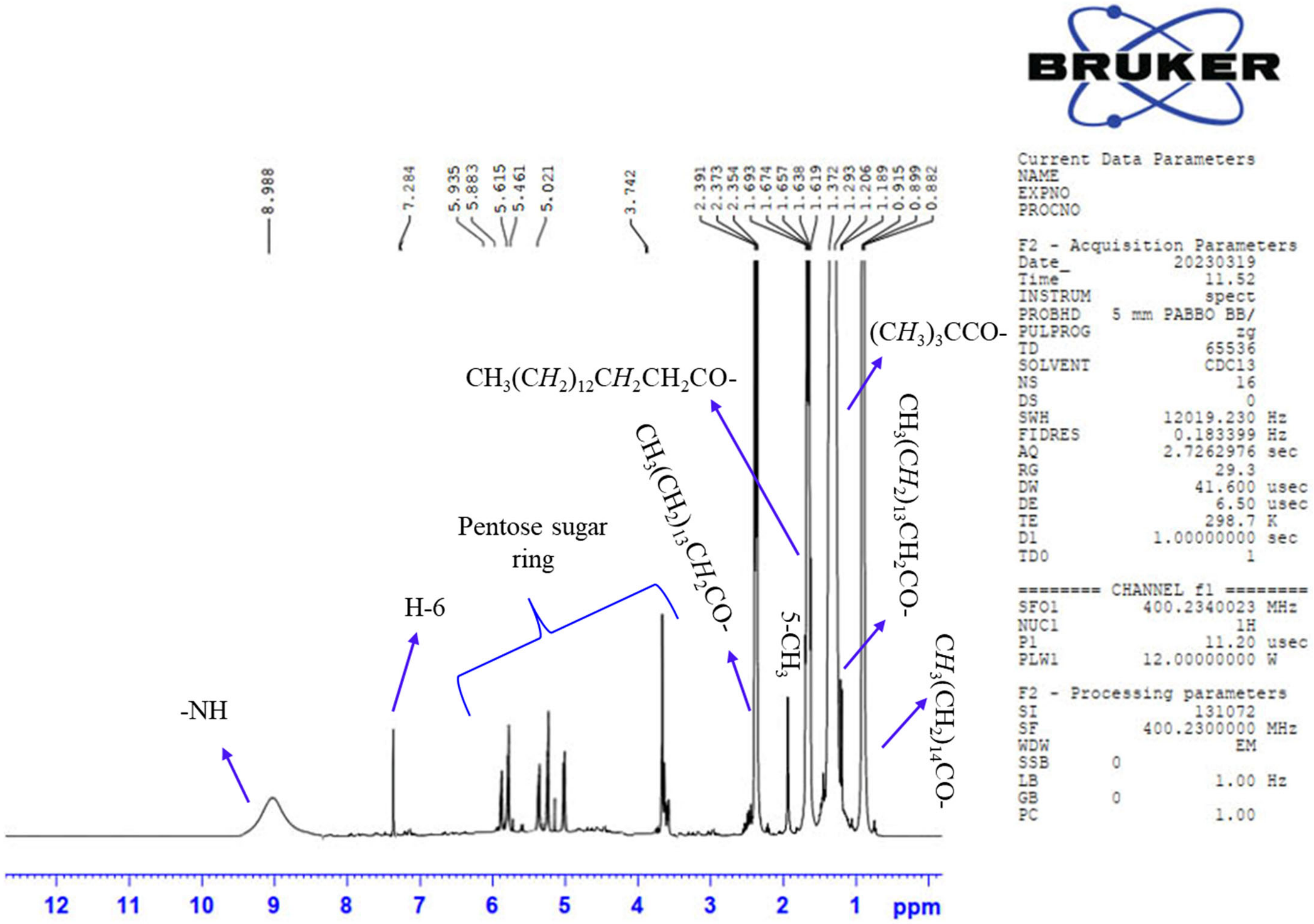

Figure S3. ^{1}H-NMR spectra of the compound (**3**).

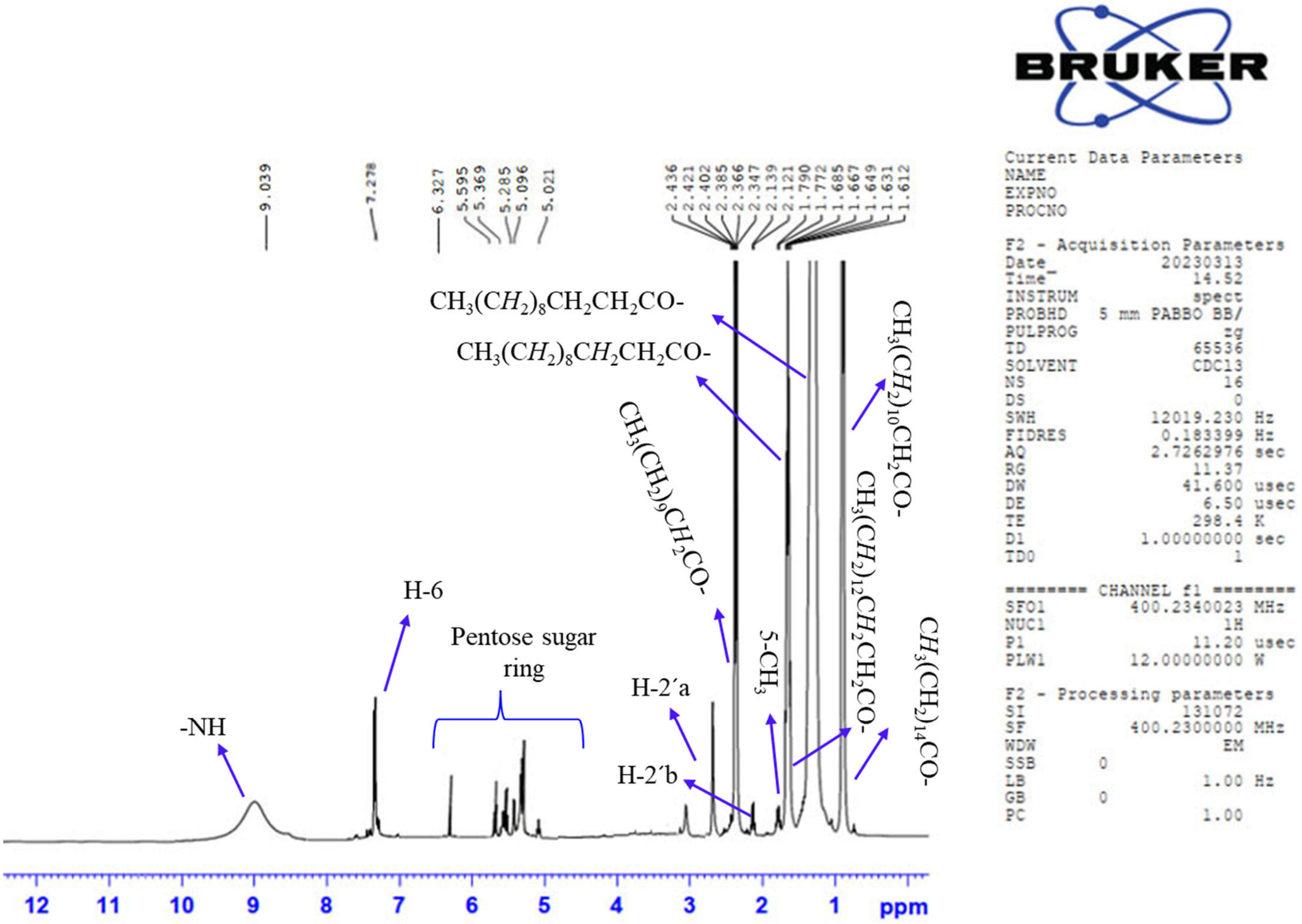

Figure S4. ^{1}H-NMR spectra of the compound (**4**).

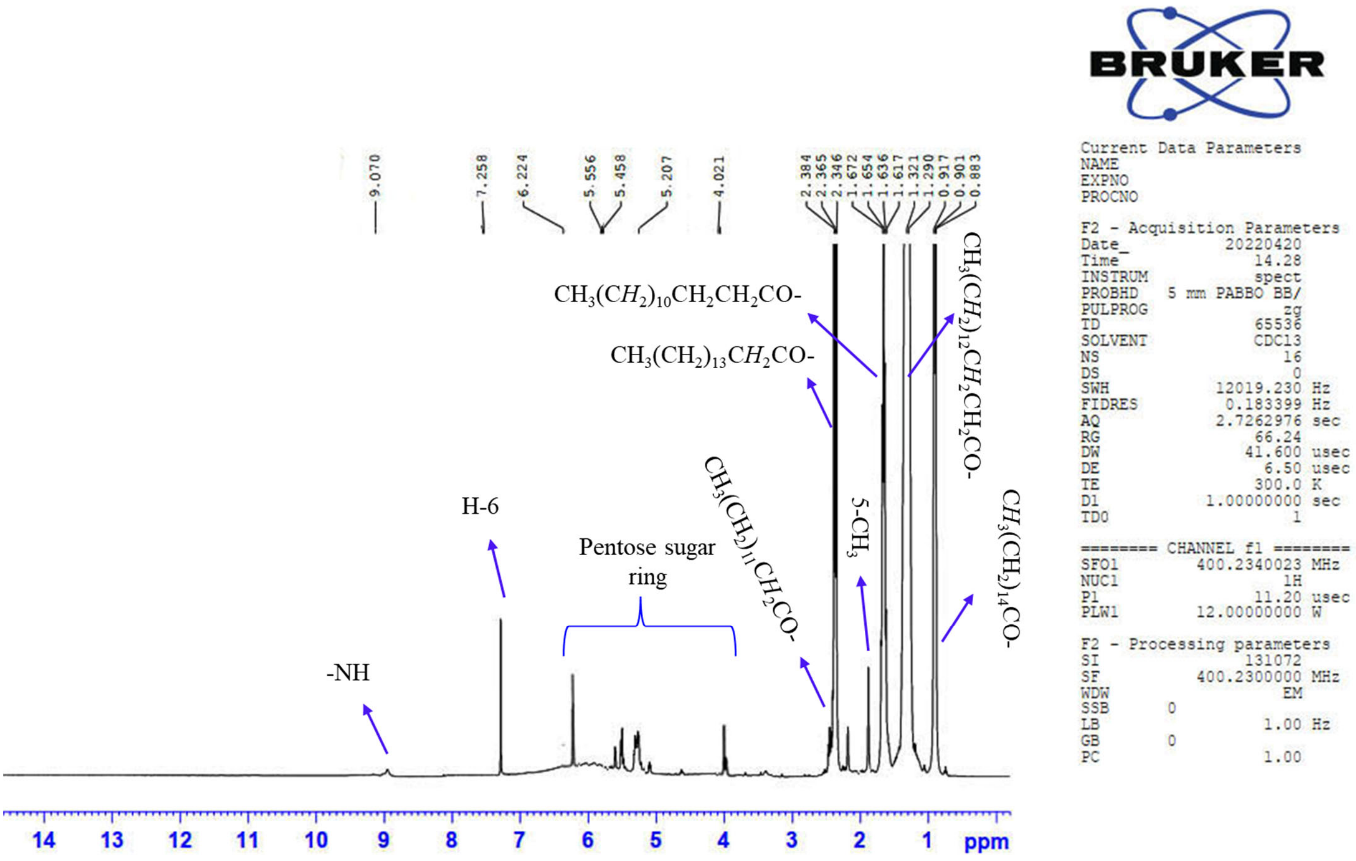

Figure S5. ^{1}H-NMR spectra of the compound (**5**).

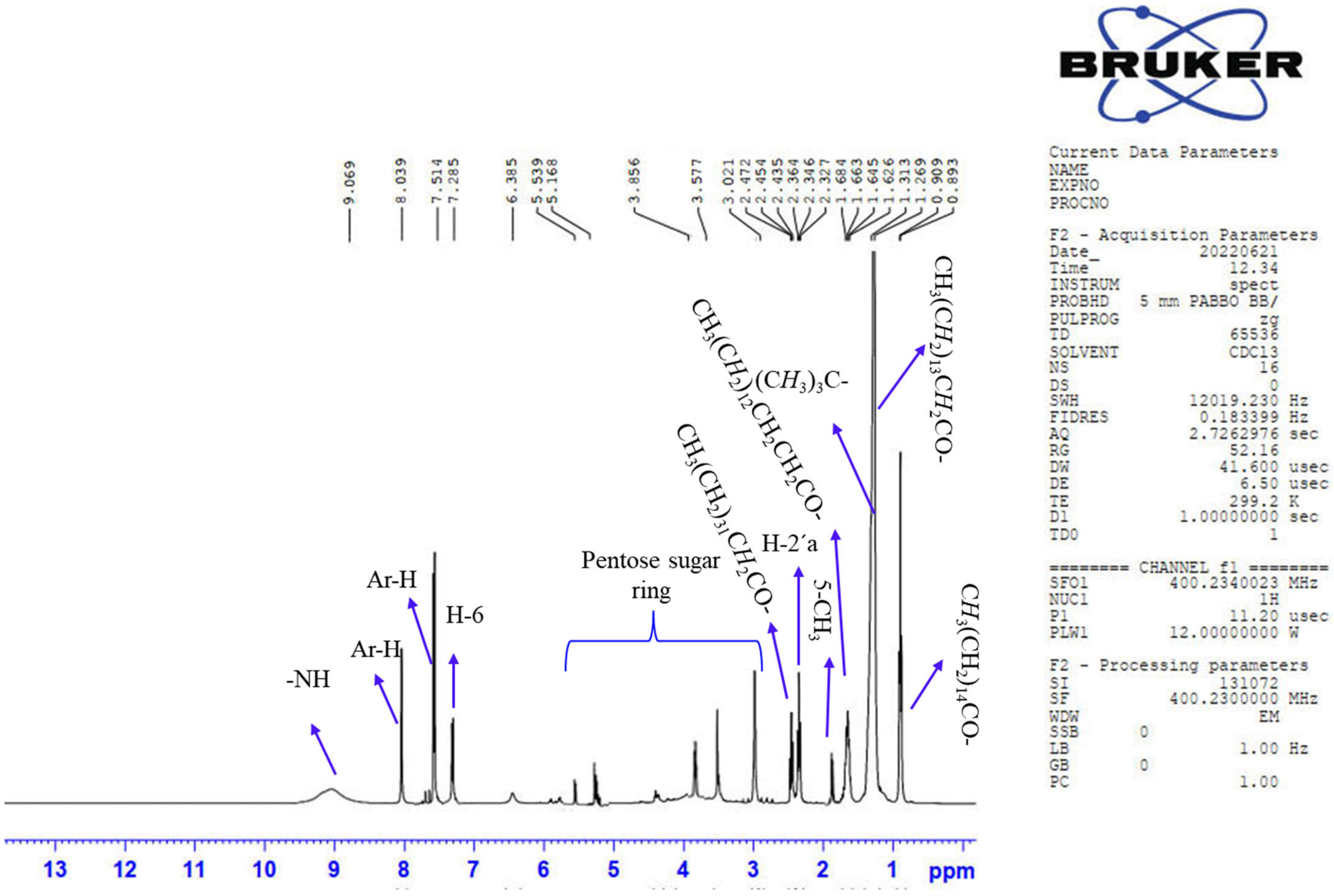

Figure S6. ^{1}H-NMR spectra of the compound (**6**).

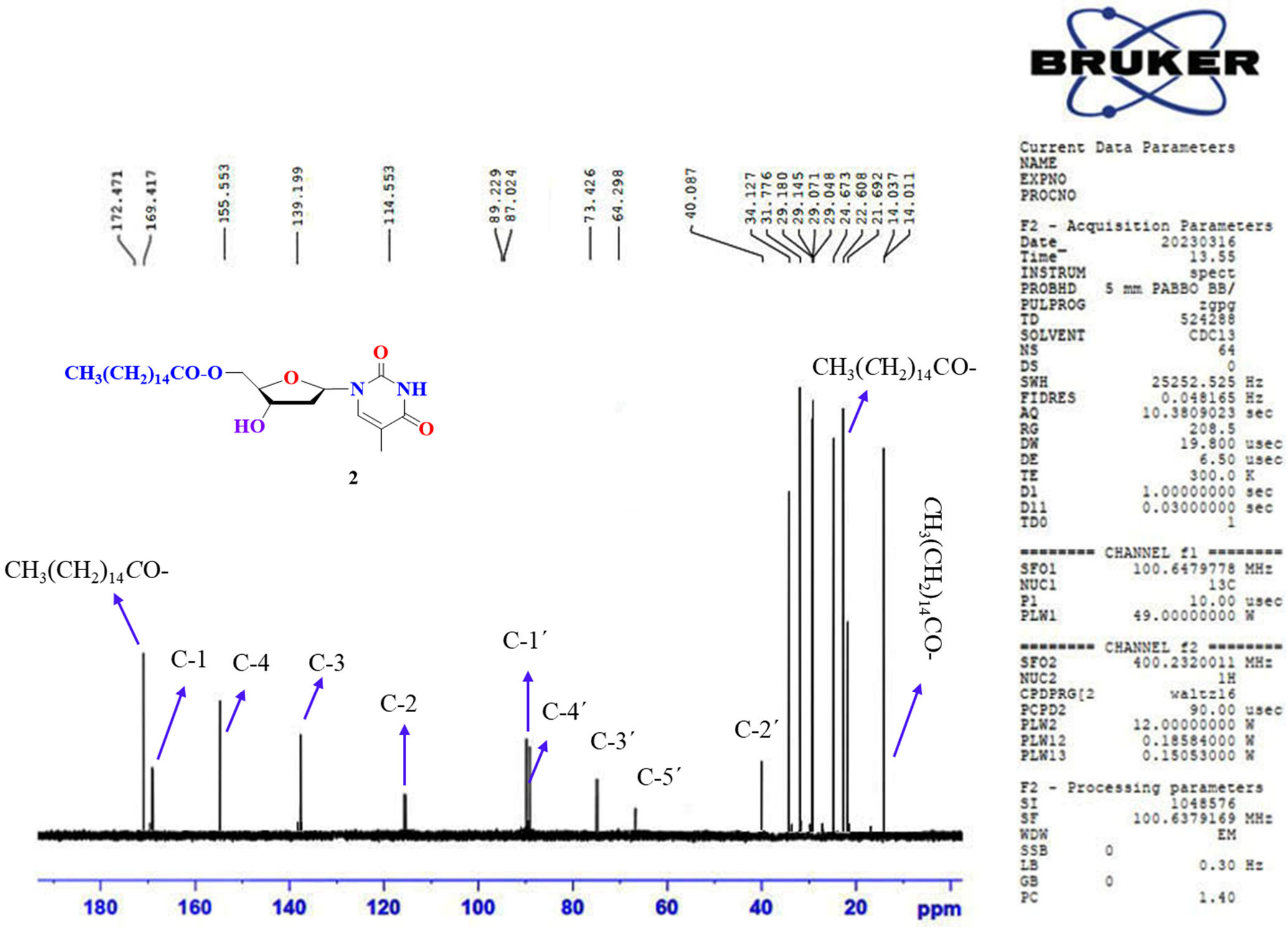

Figure S7. ^{13}C-NMR spectra of the compound (**2**).

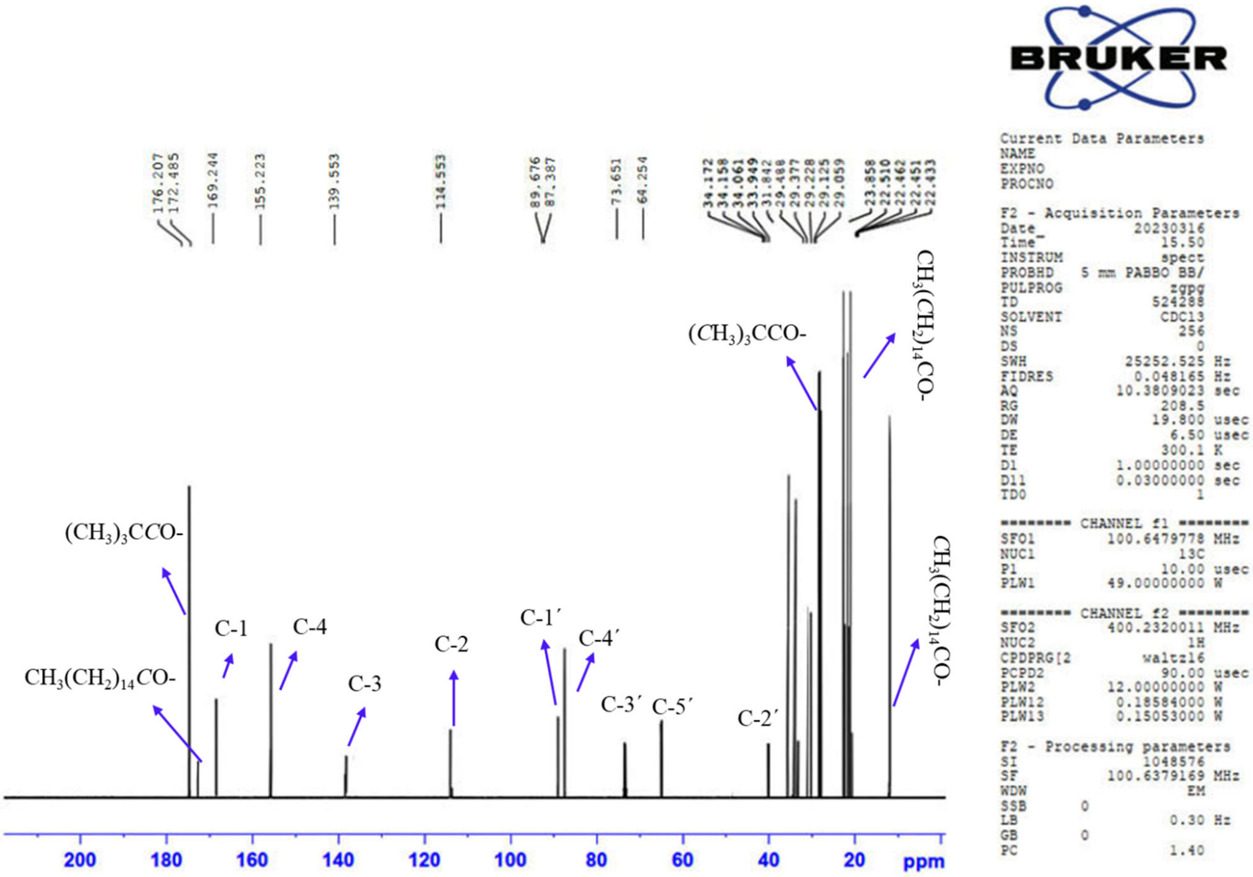

Figure S8. ^{13}C-NMR spectra of the compound (**3**).

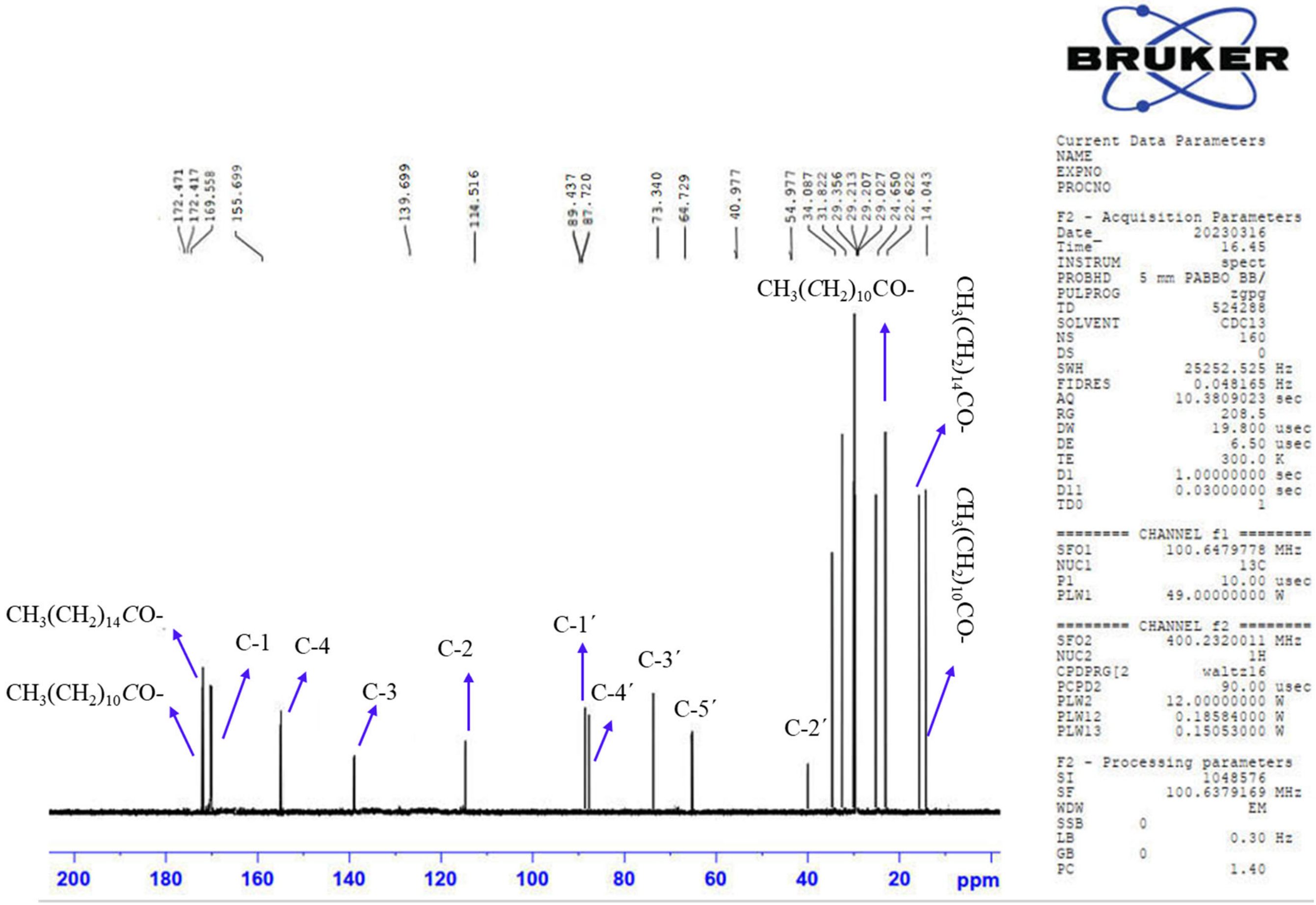

Figure S9. ^{13}C-NMR spectra of the compound (**4**).

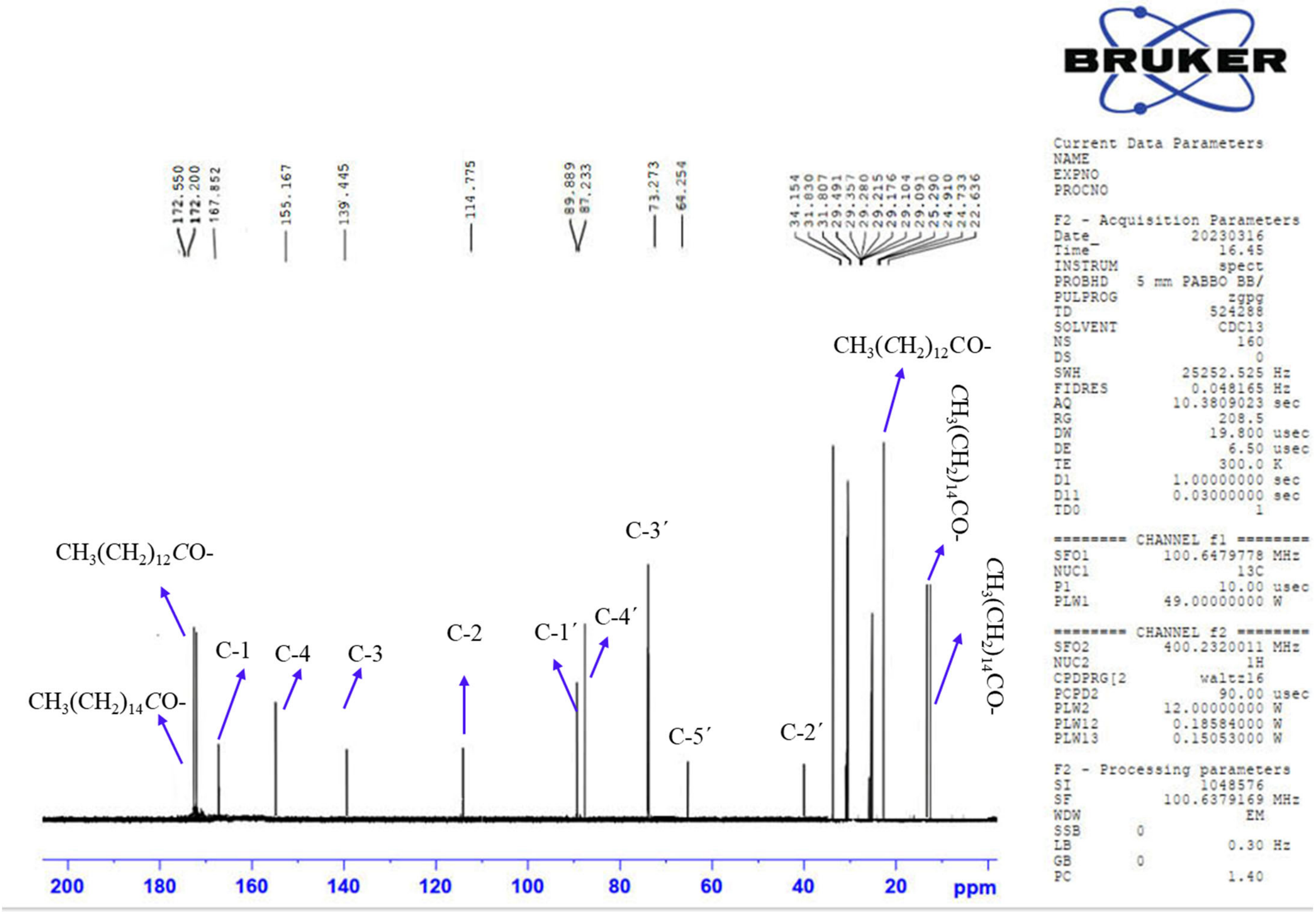

Figure S10. ^{13}C-NMR spectra of the compound (**5**).

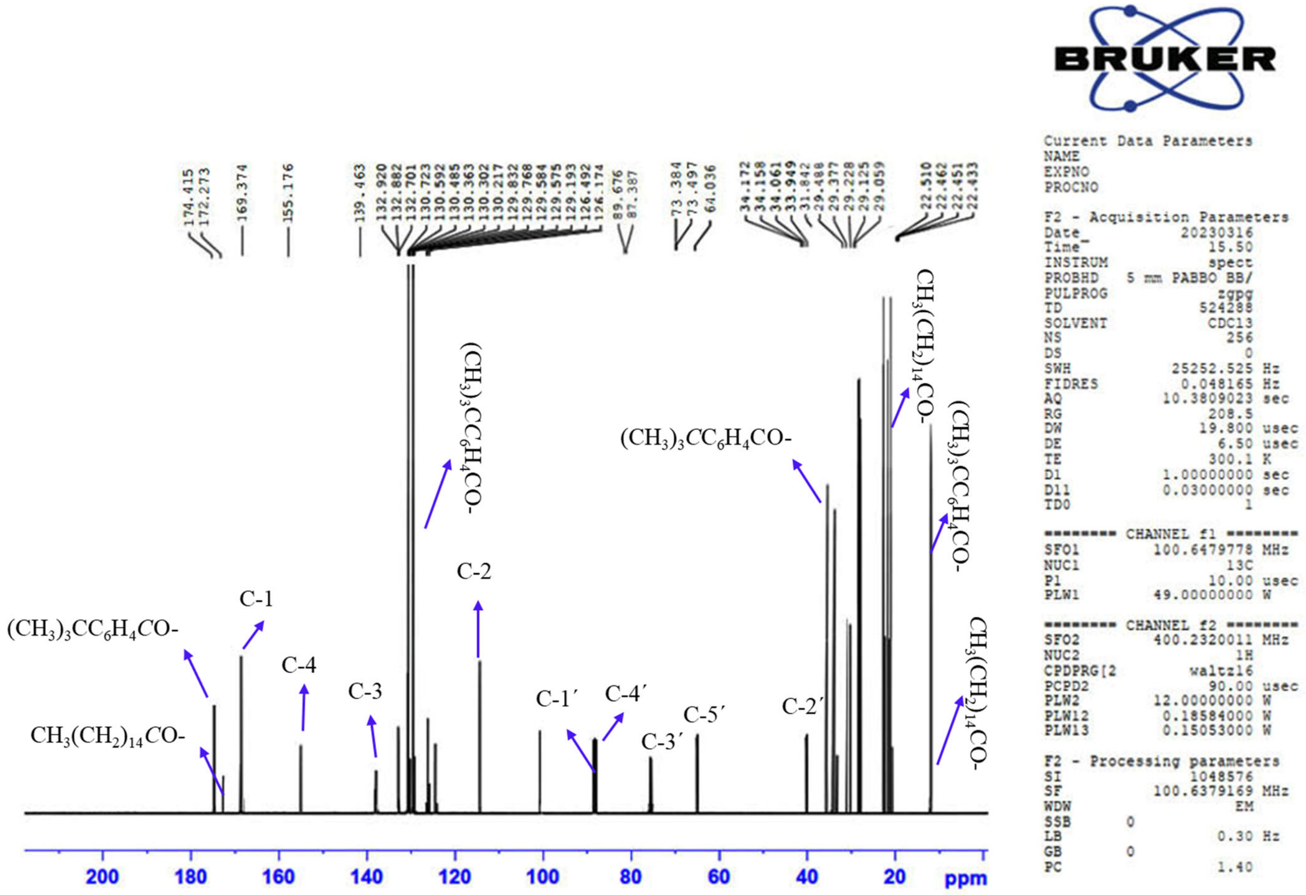

Figure S11. ^{13}C-NMR spectra of the compound (**6**).

3. Biological data

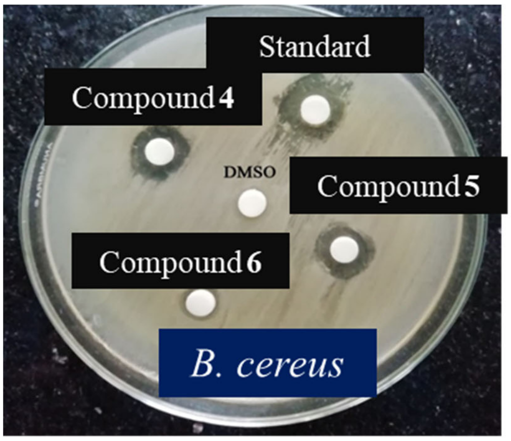

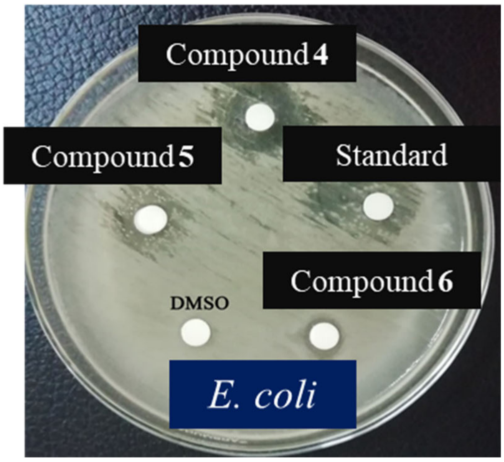

Figure S12. Experimental dishes of the synthesized test compounds **4, 5** and **6** against (**left**); *B. cereus* and (**right**); *E. coli*, Here DMSO = Negative control and Azithromycin = Positive control.

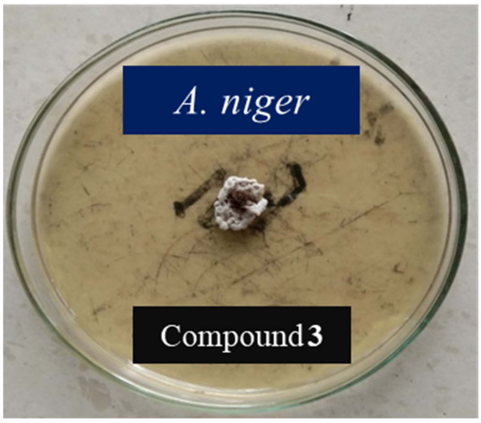

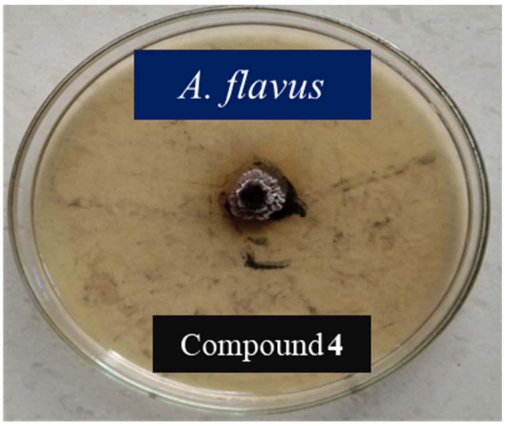

Figure S13. Mycelial growth of the synthesized compound **3** against (**left**); *A. niger* and (**right**); *A. flavus*.

4. Tables

Table S1

Name of the pathogenic microorganisms.

Types of organisms	Strain	Reference
Gram-positive bacteria	*Bacillus subtilis*	ATCC 6633
	Bacillus cereus	BTCC 19
Gram-negative bacteria	*Escherichia coli*	ATCC 8739
	Salmonella typhi	AE 14612
	Pseudomonas aeruginosa	ATCC 9027
Name of the fungi	*Aspergillus niger*	ATCC 16404
	Aspergillus flavus	ATCC 204304

Table S2

The MIC values in mg/L of compounds **4** and **5** against tested organisms.

	B. subtilis	***S. aureus***	***E. coli***	***S. typhi***	***P. aeruginosa***
**4**	0.45	6	8	1	8
**5**	2	6	1	0.5	8
Azithro	3	7	10	2	10

Table S3

The MBC values in mg/L of compounds **4** and **5** against tested organisms.

	B. subtilis	***S. aureus***	***E. coli***	***S. typhi***	***P. aeruginosa***
**4**	8	16	16	8	16
**5**	8	16	12	8	8
Azithro	10	20	14	12	12
